# Epstein-Barr virus subverts mevalonate and fatty acid pathways to promote infected B-cell proliferation and survival

**DOI:** 10.1371/journal.ppat.1008030

**Published:** 2019-09-13

**Authors:** Liang Wei Wang, Zhonghao Wang, Ina Ersing, Luis Nobre, Rui Guo, Sizun Jiang, Stephen Trudeau, Bo Zhao, Michael P. Weekes, Benjamin E. Gewurz

**Affiliations:** 1 Graduate Program in Virology, Division of Medical Sciences, Harvard Medical School, Boston, Massachusetts, United States of America; 2 Division of Infectious Diseases, Department of Medicine, Brigham and Women’s Hospital, Boston, Massachusetts, United States of America; 3 Department of Microbiology, Harvard Medical School, Boston, Massachusetts, United States of America; 4 Broad Institute of Harvard and MIT, Cambridge, Massachusetts, United States of America; 5 Department of Laboratory Medicine, West China Hospital, Sichuan University, Chengdu, Sichuan, People’s Republic of China; 6 Cambridge Institute for Medical Research, University of Cambridge, Cambridge, United Kingdom; University of Washington, UNITED STATES

## Abstract

Epstein-Barr virus (EBV) causes infectious mononucleosis and is associated with multiple human malignancies. EBV drives B-cell proliferation, which contributes to the pathogenesis of multiple lymphomas. Yet, knowledge of how EBV subverts host biosynthetic pathways to transform resting lymphocytes into activated lymphoblasts remains incomplete. Using a temporal proteomic dataset of EBV primary human B-cell infection, we identified that cholesterol and fatty acid biosynthetic pathways were amongst the most highly EBV induced. Epstein-Barr nuclear antigen 2 (EBNA2), sterol response element binding protein (SREBP) and MYC each had important roles in cholesterol and fatty acid pathway induction. Unexpectedly, HMG-CoA reductase inhibitor chemical epistasis experiments revealed that mevalonate pathway production of geranylgeranyl pyrophosphate (GGPP), rather than cholesterol, was necessary for EBV-driven B-cell outgrowth, perhaps because EBV upregulated the low-density lipoprotein receptor in newly infected cells for cholesterol uptake. Chemical and CRISPR genetic analyses highlighted downstream GGPP roles in EBV-infected cell small G protein Rab activation. Rab13 was highly EBV-induced in an EBNA3-dependent manner and served as a chaperone critical for latent membrane protein (LMP) 1 and 2A trafficking and target gene activation in newly infected and in lymphoblastoid B-cells. Collectively, these studies identify highlight multiple potential therapeutic targets for prevention of EBV-transformed B-cell growth and survival.

## Author summary

EBV, the first human tumor virus identified, persistently infects >95% of adults worldwide. Upon infection of small, resting B-lymphocytes, EBV establishes a state of viral latency, where viral oncoproteins and non-coding RNAs activate host pathways to promote rapid B-cell proliferation. EBV’s growth-transforming properties are closely linked to the pathogenesis of multiple immunoblastic lymphomas, particularly in immunosuppressed hosts. While EBV oncogenes important for B-cell transformation have been identified, knowledge remains incomplete of how these EBV factors remodel cellular metabolism, a hallmark of human cancers. Using a recently established proteomic map of EBV-mediated B-cell growth transformation, we found that EBV induces biosynthetic pathways that convert acetyl-coenzyme A (acetyl-CoA) into isoprenoids, steroids, terpenoids, cholesterol, and long-chain fatty acids. Viral nuclear antigens cooperated with EBV-activated host transcription factors to upregulate rate-limiting enzymes of these biosynthetic pathways. The isoprenoid geranylgeranyl pyrophosphate was identified as a key product of the EBV-induced mevalonate pathway. Our studies highlighted GGPP roles in Rab protein activation, and Rab13 was identified as a highly EBV-upregulated GTPase critical for LMP1 and LMP2A trafficking and signaling. These studies identify multiple EBV-induced metabolic enzymes important for B-cell transformation, including potential therapeutic targets.

## Introduction

The gamma-herpes virus Epstein-Barr virus (EBV) causes infectious mononucleosis (IM) and is associated with multiple B-cell and epithelial malignancies [1]. EBV is a major source of B-cell lymphoproliferative disease in immunosuppressed hosts, including following organ transplantation, with human immunodeficiency virus co-infection, with immunosenescence of aging or in the setting of primary immunodeficiency [2–4]. EBV causes endemic Burkitt’s lymphoma (BL), the most common pediatric lymphoma in sub-Saharan Africa [5, 6] and is strongly associated with a subset of Hodgkin’s lymphoma (HL) [1, 7]. While much has been learned about viral factors necessary for oncogenic transformation, knowledge remains incomplete of how EBV manipulates host metabolic pathways, a hallmark of cancer [8].

In primary infection, EBV translocates across the tonsillar epithelial barrier to reach the B-cell compartment, which is the reservoir for lifelong infection. Whereas epithelial cell infection typically results in production of infectious virions by the viral lytic cycle, EBV establishes latency in newly infected B-cells. Early in the course of IM, considerable numbers of latently-infected B-cells can be detected in peripheral blood [9], although innate and adaptive immune responses subsequently limit the outgrowth of cells that express viral transforming proteins [10, 11].

EBV uses a series of latency programs to expand the infected cell reservoir and to reach the memory B-cell compartment, the site of long-term latency [1, 12]. In vitro, EBV has the remarkable ability to convert resting primary B-cells into immortalized lymphoblastoid cell lines (LCLs). Reverse genetics identified Epstein-Barr nuclear antigens (EBNAs) and latent membrane protein (LMPs) necessary for in vitro B-cell transformation [13], but much remains to be learned about how these factors achieve B-cell growth transformation. Importantly, the transition from B-cell quiescence to rapid proliferation requires major metabolic pathway remodeling, only some of which are presently understood.

EBV B-cell growth transformation takes place over at least three phases [14]. Over the first 72 hours of infection, the EBV-encoded transcription factors EBNA2 and EBNA-leader protein (EBNA-LP) are highly expressed as EBV converts quiescent B-lymphocytes into activated blasts. EBNA2 uses RBP-Jκ and other B-cell transcription factors to reach host and viral genome sites. EBNA2 highly upregulates the proto-oncogene MYC [15–17], which together with its binding partner MAX can strongly influence metabolic remodeling [18]. However, key EBNA2, EBNA-LP and MYC target genes important for primary B-cell remodeling and activation remain to be defined in this early period post-infection.

Over days 3 to 7 post-infection, viral oncogenes, together with their host targets, drive rapid BL-like cell proliferation, with mitosis occurring as frequently as every 8–12 hours [14]. Over this period, EBNA2 upregulates EBNA3 and LMPs [19–24] as EBV remodels many host pathways. These include upregulation of glycolysis, oxidative phosphorylation and the mitochondrial one-carbon pathway [25, 26], though knowledge of EBV-mediated metabolic pathway remodeling in support of hyperproliferation remains incomplete. EBV-driven increases in amino acid uptake may also activate the nutrient sensing mammalian target of rapamycin (mTOR) pathway [27, 28] in this phase of B-cell transformation. By day 7 post-infection, infected cells begin to demonstrate lymphoblastoid-like cell physiology as LMP1 and 2A signaling increases and cell division slows to once daily [14, 25, 29, 30]. LMP1 and LMP2A mimic CD40 and B-cell receptor signaling to activate NF-κB and PI3K/AKT/mTOR pathways, respectively [31–34]. Host factors important for LMP trafficking to membrane lipid raft signaling sites remains incompletely described.

Remodeling and rapid cell growth place huge demands on lipid and fatty acid biosynthesis. We therefore used a recently constructed proteomic map to gain insights into EBV-driven metabolic pathway remodeling, where multiplex tandem mass tag mass spectrometry was performed on whole cell and plasma membrane fractions of primary human B-cells at rest and at nine timepoints following B95-8 EBV strain infection [26]. To increase dataset robustness, this proteomic analysis was performed in biological triplicate, using primary B-cells from four human donors for each replicate. Consistent with prior reports, proteomic analysis identified that EBV highly induces glucose uptake and aerobic glycolysis in newly infected B-cells [25, 28, 29]. Although glycolysis generates less ATP per metabolized glucose, it together with downstream pathways such as the TCA cycle produce intermediates for anabolic reactions.

Here, we identified that EBV highly induces the mevalonate pathway, which uses glucose-derived acetyl-CoA and NADPH to produce sterols, isoprenoids, cholesterol and fatty acid synthesis in newly infected B-cells. We then investigated how EBV and host transcription factors activate these key anabolic pathways and identified unexpected downstream roles in EBV-driven B-cell proliferation and survival.

## Results

### EBV highly induces cholesterol and fatty acid synthesis pathways in newly infected B-cells

**Fig 1:**
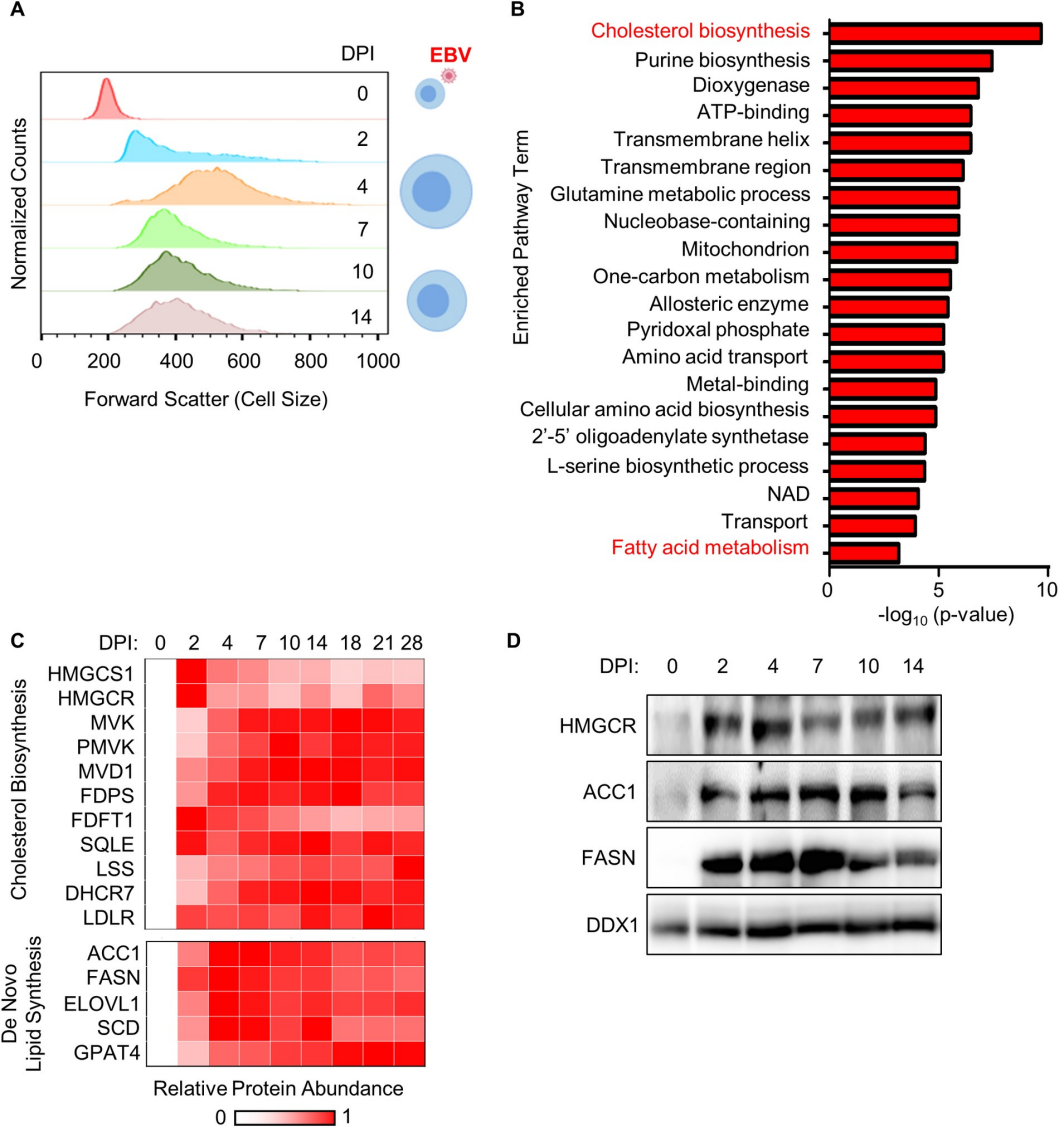
EBV highly upregulates lipid biosynthesis programs in newly infected primary B-cells undergoing cell remodeling. (A) FACS forward scatter cell size measurements of CD19+ primary human B-cells at the indicated days post-infection (DPI) by EBV. Cells were gated for CD23 positivity at timepoints after day 0 to identify EBV-infected B-cells [135–137]. See also S1 Fig. (B) Pathway enrichment analysis of proteins whose abundance was at least 2-fold upregulated by EBV infection at 4 DPI versus resting B-cell levels and with p<0.075 across three biological replicates in our proteomic analysis (28). Only proteins with annotated metabolic function identified in a recent curated dataset (36) were used for bioinformatic analysis. (C) Heatmap representation of proteomic cholesterol and de novo lipid biosynthesis pathway enzyme relative abundances at the indicated DPI of primary human B cell infection by EBV. See also S2 Fig. (D) Immunoblot analysis of HMGCR, ACC1, FASN and load-control DDX1 levels in whole cell lysate (WCL) prepared from primary B-cells infected by EBV for the indicated days. Representative blots (n = 3) are shown.

EBV infection causes dramatic B-cell remodeling that begins early after infection and converts resting B-cells into activated lymphoblasts. Underscoring the need for anabolic pathway upregulation, EBV infection significantly increased cell size over the first week post-infection. Flow cytometry (FACS) analysis identified that newly EBV-infected primary B-cells reach a maximum size at 4 days post-infection (DPI) (Figs 1A and S1), consistent with a recent publication [35]. EBV-induced increases in cell size were evident in cells at multiple stages of the cell-cycle (S1 Fig). To gain insights into metabolic pathways highly upregulated upon EBV infection, enrichment analysis was performed on our recently generated multiplexed tandem mass tag proteomic analysis of newly EBV infected primary human B-cells [28]. Using a curated list of human metabolism-associated proteins [36], the cholesterol biosynthesis pathway was found to be the most highly-upregulated host biosynthetic pathway at 96 hours post-EBV infection versus resting B-cell baseline levels (Fig 1B and 1C, S1 and S2 Tables). Notably, the enzyme HMGCS1, which condenses acetoacetyl-CoA and acetyl-CoA to produce 3-hydroxy-3-methylglutaryl-CoA (HMG-CoA), and the rate-limiting pathway enzyme HMG-CoA reductase (HMGCR) [37] were strongly upregulated even by 2 days post-infection (DPI), prior to the onset of cell proliferation. EBV induction of HMGCR was validated by immunoblot (Fig 1D).

EBV infection induced the eight enzymes that convert mevalonate into cholesterol (Fig 1C). EBV also rapidly upregulated whole cell and plasma membrane levels of the low-density lipoprotein receptor (LDLR), which mediates endocytosis of cholesterol-rich LDL [38, 39] (Figs 1C, S2A and S2B). Sustained expression of cholesterol biosynthetic enzymes and LDLR is suggestive of an ongoing role in the lymphoblastoid phase; in further support, LCLs abundantly express LDLR [40].

Further underscoring key host lipid metabolism roles in early EBV-driven B-cell growth transformation, enrichment analysis also highlighted fatty acid metabolism as highly EBV-induced (Fig 1B). EBV upregulated the rate limiting fatty acid biosynthesis pathway enzyme acetyl-CoA carboxylase 1 (ACC1), which converts acetyl-CoA into malonyl-CoA. EBV also induced the subsequent pathway enzyme fatty acid synthase (FASN), which converts malonyl-CoA to palmitate, an important substrate for palmitoylation, triglyceride and long chain fatty acid production [41] (Figs 1C and S2C). ACC1 and FASN upregulation were validated by whole cell lysate immunoblot analysis (Fig 1D). Of note, DDX1 was used as load controls, since its protein abundance remained relatively unchanged during the EBV infection time course (S2D Fig).

### SREBP, EBNA2 and MYC induce cholesterol and fatty acid biosynthesis enzymes

**Fig 2:**
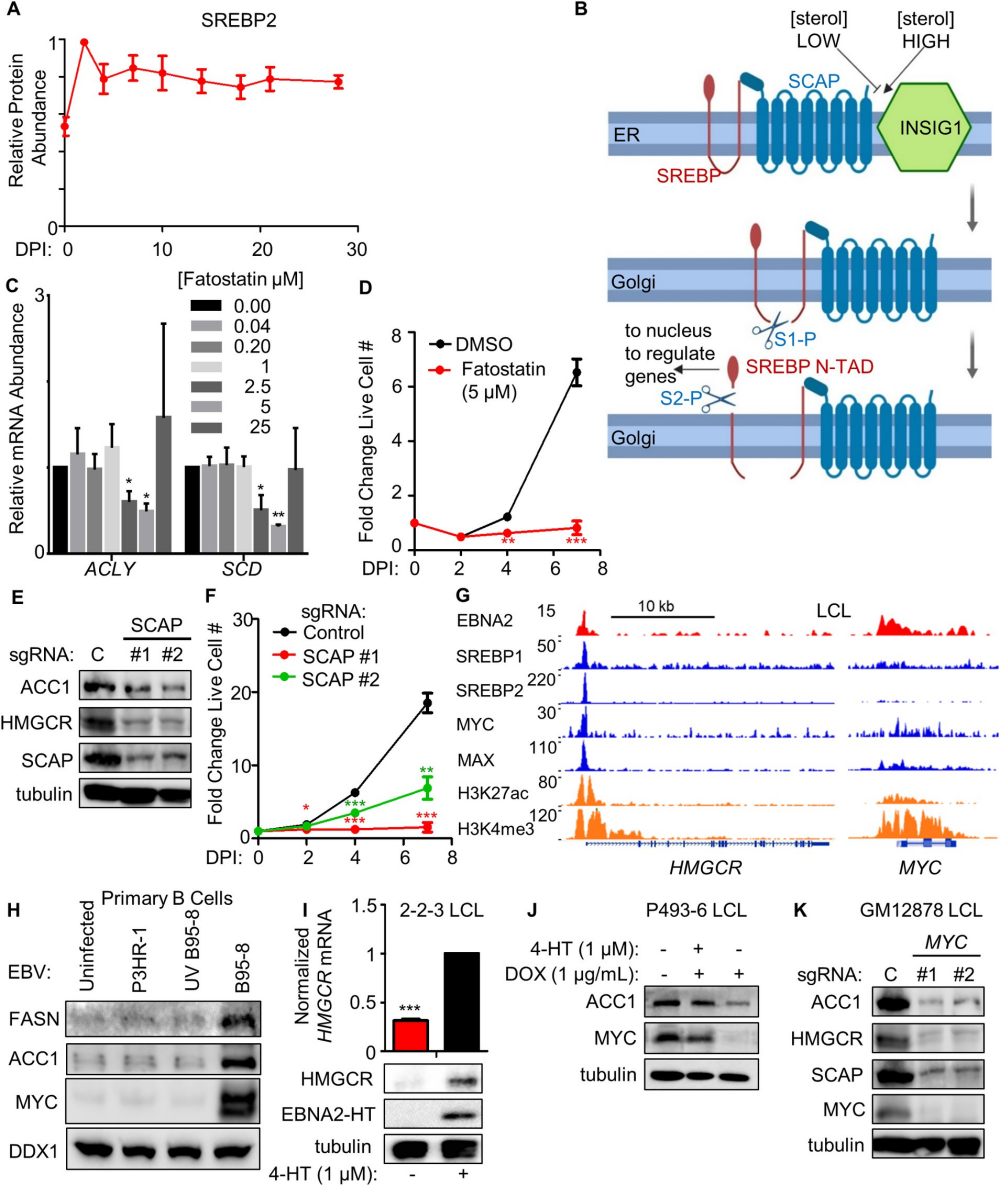
EBNA2, MYC and SREBPs induce key lipid biosynthesis enzymes. (A) Temporal profile of SREBP2 protein abundance over the indicated days post-infection (DPI) of primary human B cells. Data show the mean + SEM from three biological replicates, n = 3. (B) Schematic showing SREBP regulation by sterol levels. Under sterol-sufficient conditions, SCAP binds to INSIG1 to promote SREBP ER retention. When sterol levels are insufficient, SCAP dissociates from INSIG1 and chaperones SREBPs to the Golgi apparatus, where site-1 and site-2 proteases (S1-P and S2-P, respectively) cleave SREBPs. Transcriptionally active N-terminal SREBP domains (denoted as ‘N-TAD’) re-localize to the nucleus to transactivate cholesterogenic and lipogenic target genes. (C) Quantitative RT-PCR analysis of SREBP target gene *ACLY* and *SCD* mRNA abundances in primary human B-cells infected by B95-8 EBV which were cultured in the presence of DMSO or the indicated SREBP/SCAP inhibitor fatostatin doses for days 2–7. Data show the mean + SEM fold change values for n = 3 replicates, *, p<0.05, **, p<0.01 (two-tailed t-test). (D) Growth curve analysis of newly infected primary human B-cells cultured in the presence of DMSO or fatostatin at 5 μM from days 2–7. Data show the mean + SEM fold change values relative to day 0, n = 3. **, p<0.01; ***, p<0.005 (two-tailed t-test). See also S3D Fig. (E) Immunoblot analysis of ACC1, HMGCR, SCAP and tubulin levels in WCL prepared from Cas9+ GM12878 LCL expressing the indicated non-targeting control (C) or independent *SCAP* targeting sgRNAs. Representative blots of n = 3 replicates are shown. (F) Growth curve analysis of Cas9+ GM12878 LCL that express either control or independent SCAP-targeting sgRNAs. Mean + SEM fold change values relative to day 0 levels are shown for n = 3 replicates. *, p<0.05; **, p<0.01; ***, p<0.005 (two-tailed t-test). (G) Chromatin immunoprecipitation (ChIP)-sequencing (ChIP-seq) tracks for the indicated transcription factors or histone epigenetic marks histone 3 lysine 27 acetyl (H3K27Ac) or histone 3 lysine 4 trimethyl (H3K4Me3) at the LCL *HMGCR* locus. Shown to the right for comparison are tracks for the well characterized EBNA2 target *MYC* as a positive control. Y-axis ranges are indicated for each track. (H) Immunoblot analysis for the indicated B-cell proteins using WCL obtained from primary cells that were either mock-infected or infected with equal amounts of the non-transforming P3HR-1, UV-irradiated B95-8 or B95-8 EBV strains for four days. Representative blots of n = 2 replicates are shown. See also S3F Fig. (I) Quantitative PCR analysis and immunoblot analysis of samples prepared from EBNA2-HT LCLs grown in the absence (EBNA2 non-permissive) or presence (EBNA2 permissive) of 4HT (1 μM) for 48 hours. Quantitative PCR data show the mean + SEM from n = 3 replicates. ***, p<0.005 (one-sample t-test). Representative blots (n = 3) are shown. (J) Immunoblot analysis of WCL prepared from conditional P493-6 LCLs treated with doxycycline (DOX) to suppress exogenous *MYC* allele expression and/or with 4HT to induce EBNA2 activity for 48 hours, as indicated. Representative blots (n = 3) are shown. (K) Immunoblot analysis of WCL prepared from Cas9+ GM12878 LCL expressing control (C) or independent *MYC*-targeting sgRNAs, as indicated. Representative blots from n = 3 experiments are shown.

Sterol response element binding proteins (SREBPs) are basic-helix-loop-helix leucine zipper transcription factors that bind to DNA sterol response elements to induce expression of mevalonate and fatty acid pathway components [42–44]. Since SREBPs are major inducers of cholesterol and lipid metabolism programs in many contexts [45–47], we hypothesized that EBV may subvert SREBPs to transactivate cholesterol and lipid synthesis genes in newly infected B-cells. In support, SREBP2 abundance was upregulated by 2 DPI (Fig 2A), though curiously, SREBP1 was not detected by our proteomic analysis. LCL ChIP-seq identified EBNA2 occupancy of the *SREBF2* promoter (S3A Fig) [48, 49]. Notably, all four CRISPR single guide RNAs (sgRNAs) against *SREBF2*, which encodes SREBP2, were strongly depleted in our recent 21-day Cas9+ GM12878 LCL Achilles’ heel screen, but not in EBV+ BL cells [17] (S3B Fig), suggesting an important SREBP2 role in LCL growth and/or survival. By contrast, sgRNAs targeting *SREBF1*, which encodes SREBP1, were virtually unchanged in both GM12878 and P3HR-1 BL over the 21-day screen (S3C Fig).

SREBPs are also subject to tight post-translational regulation, though specific mechanisms operative in EBV-infected B-cells have not been studied. When cholesterol and lipid pools are sufficient, SREBP cleavage activating protein (SCAP) retains SREBPs at the endoplasmic reticulum (ER) by binding to the ER retention factor insulin-induced gene 1 (INSIG1) [50, 51]. Upon depletion of cholesterol or lipid pools, SREBPs traffic to the Golgi, where resident site-1 and site-2 proteases target SREBPs to liberate an N-terminal transcription factor fragment that traffics to the nucleus to regulate target genes [52] (Fig 2B).

To investigate potential SCAP roles in EBV-mediated SREBP activation, newly infected cells were treated with the small molecule antagonist fatostatin, which blocks SCAP ER-to-Golgi transport [52]. Dose response analysis established that 5 μM fatostatin achieved maximal inhibition of prototypic SREBP target genes *ACLY* and *SCD* in newly-infected primary human B-cells, to a similar extent as reported in other cell types, where the 5 μM dose was also used [52]. *ACLY* encodes ATP citrate lyase, the main enzyme responsible for cytosolic acetyl-CoA synthesis, whereas *SCD* encodes the fatty acid biosynthesis enzyme stearoyl-CoA desaturase. 5 μM fatostatin treatment, a dose that was very close to the empirically established half maximal effective concentration (EC_50_), significantly impaired EBV-mediated primary B-cell outgrowth (Figs 2C, 2D and S3D). Together, these results suggest that on-target fatostatin activity underlies its effect on EBV-mediated primary B-cell outgrowth.

To next test SCAP LCL roles, control versus independent *SCAP-*targeting sgRNAs were expressed in Cas9+ GM12878 LCLs. SCAP depletion strongly downregulated LCL HMGCR and ACC1 expression and LCL proliferation (Fig 2E and 2F), suggesting shared SCAP roles in primary B-cell and fully transformed LCL SREBP-mediated target gene regulation. Further suggestive of SREBP2 roles in EBV-transformed cells, GM12878 ChIP-seq [48, 49] identified SREBP2 occupancy at the *HMGCR* promoter (Fig 2G), and *SREBF2* mRNA is expressed in all of the >200 LCLs profiled by RNA-seq [53].

LCL ChIP-seq datasets [17, 48, 49] were next used to identify host and viral additional transcription factors present at the *HMGCR* and *ACC1* promoters. Interestingly, EBNA2, RBP-Jκ, MYC and MAX were found to co-occupy *HMGCR* and *ACC1* promoter sites together with SREBP2 (Figs 2G and S3E), indicating possible shared roles in mevalonate and fatty acid pathway induction. ChIP-seq signals at the well-established EBNA2 target gene *MYC* are shown for comparison (Fig 2G). Consistent with their important regulatory roles in EBV-mediated B-cell metabolic pathway remodeling, EBNA2, EBNA-LP, MYC and MAX co-occupy the *SREBF2* LCL promoter and a site in the 1^st^ intron (S3A Fig).

To investigate whether EBNA2 and EBNA-LP are required for EBV-driven ACC1 or FASN induction, primary cells were infected with equal amounts of the non-transforming EBV strain P3HR-1, which is deficient for *EBNA2* and most of the *EBNA-LP* genes [54–57] or with transforming B95-8 virus. P3HR-1 infection failed to upregulate ACC1, FASN or EBNA2 target MYC, as did infection with UV-irradiated B95-8, which can enter cells but does not induce EBV-encoded genes [58, 59] (Fig 2H). By contrast, transforming B95-8 virus that had not been pre-treated was able to upregulate ACC1 and MYC. Similarly, B95-8 but not UV-treated B95-8 or P3HR-1 upregulated mRNA levels of SREBP2 targets *ACYL*, *SCD*, *HMGCR*, *FDFT1*, and *LDLR* (S3F Fig). These data suggest that EBNA2 and/or EBNA-LP, rather than an innate immune response to the incoming viral particle, are required for fatty acid and cholesterol biosynthetic pathway enzyme induction in newly infected B-cells.

To next investigate EBNA2 roles in sustained cholesterol and lipid biogenesis in transformed cells, we used LCLs with conditional *EBNA2* alleles. 2-2-3 and P493-6 LCLs each express an EBNA2 fusion protein (EBNA2-HT), in which EBNA2 is fused to a mutant estrogen receptor ligand-binding domain under the control of 4-hydroxytamoxifen (4HT). In the absence of 4HT, EBNA2-HT re-localizes to the cytoplasm and is destabilized [16]. Conditional EBNA2 inactivation by 4HT withdrawal for 48 hours diminished HMGCR and ACC1 abundances (Fig 2I and 2J), in support of an LCL EBNA2 role in upregulation of these rate limiting enzymes.

LCL ChIP-seq identified that the oncoproteins MYC and MAX, which form a heterodimeric complex that binds to E-box DNA sites to amplify target gene transcription [46], also co-occupied the *HMGCR* promoter (Fig 2G). To investigate MYC roles in upregulating key LCL lipid synthesis genes, we first asked whether MYC could rescue the loss of ACC1 expression in LCLs upon conditional EBNA2 inactivation. P493-6 LCLs stably express a conditional TET-off *MYC* allele, allowing for rescue of *MYC* expression in LCLs with conditional EBNA2 inactivation [60, 61]. High level MYC expression induced by doxycycline withdrawal was sufficient to drive ACC1 expression, even with EBNA2 inactivation by 4HT withdrawal, suggesting that elevated MYC expression can compensate for the absence of EBNA2 (Fig 2J). Further underscoring a MYC role in fatty acid metabolism induction in LCLs, MYC depletion by two independent sgRNAs strongly decreased Cas9+ GM12878 ACC1 and HMGCR expression (Fig 2K). Intriguingly, MYC loss also strongly downmodulated SCAP abundance (Fig 2K), suggesting a further level of cross-regulation between MYC and the SCAP-SREBP axis in LCLs.

### The mevalonate pathway product geranylgeranyl pyrophosphate is important for EBV-infected B-cell outgrowth

We next investigated the biological significance of mevalonate and cholesterol pathway induction in newly-infected cells. Notably, it was previously reported that treatment with simvastatin, but not with the related HMG-CoA reductase inhibitor pravastatin, causes LCL death [62]. This phenotype was attributed to off-target simvastatin effects on leukocyte function antigen 1 (LFA-1), an LMP1-induced integrin, and also on simvastatin-mediated dissociation of LMP1 from lipid rafts [62]. Consistent with important mevalonate pathway roles in EBV LMP activation, CRISPR *HMGCR* knockout suppressed LMP1-driven TRAF1 expression and LMP2A mediated SYK and PI3K subunit p85 phosphorylation in GM12878 LCLs (S4A Fig).

**Fig 3:**
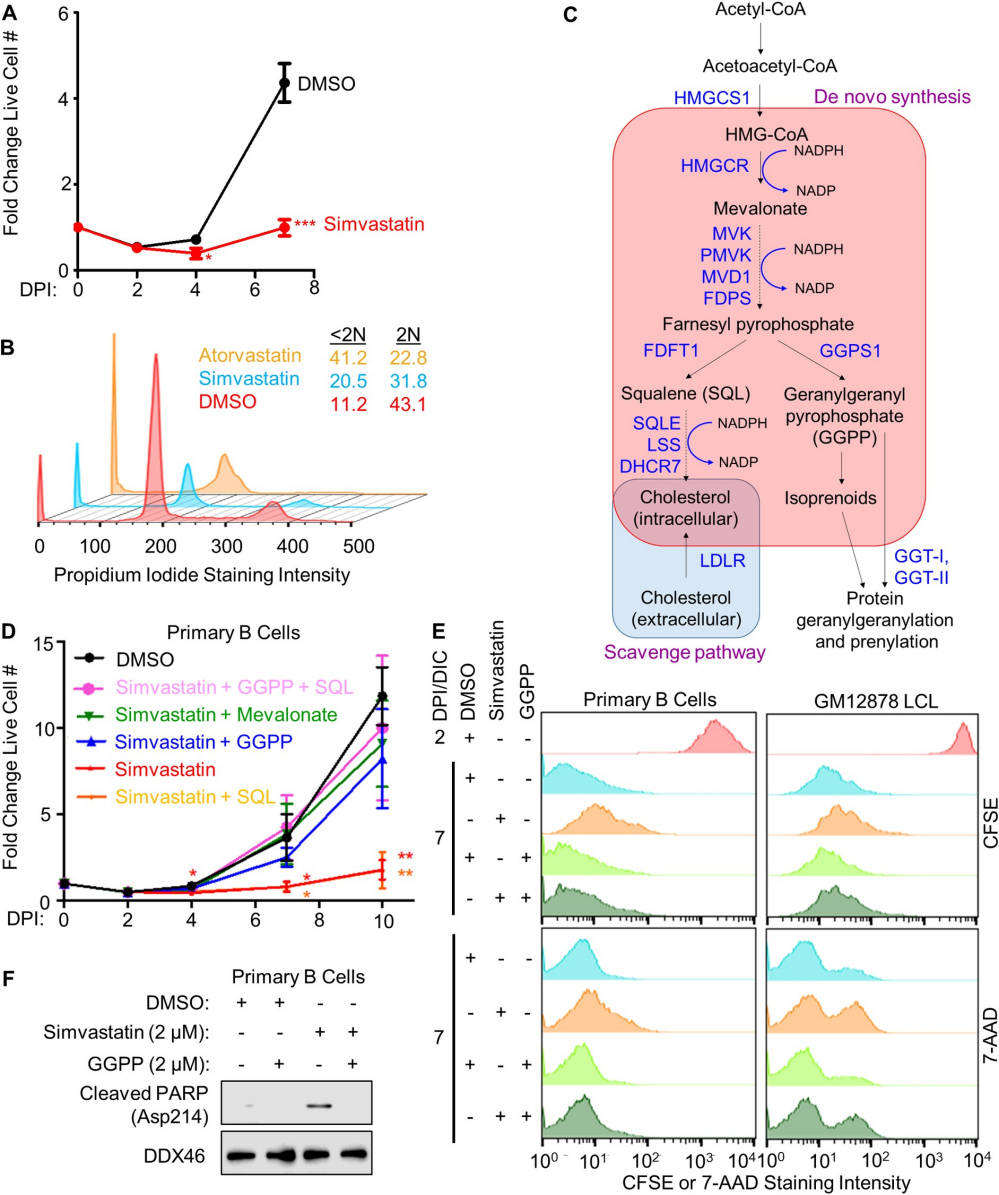
EBV-induced mevalonate metabolism supplies geranylgeranyl pyrophosphate (GGPP) for newly infected B-cell outgrowth. (A) Fold change in live cell number of primary human B-cells infected by EBV B95-8 for the indicated DPI, cultured in the presence of DMSO or simvastatin (2 μM) from 2–7 DPI. Simvastatin was refreshed at 4 DPI. Data show the mean + SEM from n = 3 replicates. *, p<0.05; ***, p<0.005 (two-tailed t-test). (B) Flow cytometric analysis of propidium iodide levels in fixed primary B-cells that had been treated with DMSO or the HMG-CoA reductase inhibitors simvastatin (2 μM) or atorvastatin (2 μM) from 2 DPI to 7 DPI. Representative cell cycle analysis plots from n = 2 experiments are shown. The percentages of cells with <2N (sub-G1) and 2N (G1) DNA content are indicated. (C) Schematic diagram of the mevalonate pathway branches that produce cholesterol or isoprenoids, with key enzymes indicated in dark blue color. Acetyl-CoA can be reduced in an NADPH-dependent manner by HMGCR to produce mevalonate. Mevalonate is then converted into farnesyl pyrophosphate, which can either be used to synthesize squalene for cholesterol biosynthesis or be diverted toward GGPP syntheses. Cholesterol can also be scavenged from the extracellular environment via the LDL receptor (LDLR). (D) Relative fold-change in live cell numbers of newly infected primary B-cells cultured in the presence of DMSO or simvastatin (2 μM) and supplemented with squalene (SQL, 100 μM), geranylgeranyl pyrophosphate GGPP (2 μM) or mevalonate (500 μM) as indicated from 2 to 7 DPI. Simvastatin, GGPP, SQL and mevalonate were refreshed at each timepoint. Data show the mean + SEM from n = 3 replicates. *, p<0.05; **, p<0.01 (two-tailed t-test). (E) Flow cytometric analysis of CFSE dye dilution proliferation and 7-AAD cell death assays in newly infected primary B-cells or GM12878 LCL. Cells were treated with DMSO, simvastatin (2 μM) and/or GGPP (2 μM), as indicated from days post-infection (DPI) 2–7, or from days in culture (DIC) 2–7 for GM12878 LCLs. Representative histograms from n = 3 replicates are shown. See also S4E Fig. (F) Immunoblot analysis of WCL from newly infected primary B-cells cultured in the presence of DMSO, simvastatin or GGPP from days 2–7 post infection. Cleaved PARP (aspartate 214 specific) was detected, together with DDX46 load-control. Representative blots from n = 3 experiments are shown.

Since newly infected primary human B-cells do not express LMP1 at levels as high as those observed in LCLs until 1 to 2 weeks post-infection [29], we first sought to determine whether early EBV-driven B-cell outgrowth was sensitive to simvastatin. Dose-response analysis in newly infected primary B-cells found that 2 μM simvastatin maximally induced the LDL receptor, a sensitive readout of statin on-target activity [63]. 1–2 μM simvastatin maximally induced cholesterol pathway enzymes FDFT1 and SQLE (S4B Fig), whose expression also have been found to increase upon HMGCR inhibition by statin drugs. Notably, 2 μM simvastatin significantly impaired EBV-driven primary B-cell outgrowth (Fig 3A), and dose response analysis identified 1.6 μM as the simvastatin EC50 (S4C Fig), suggesting on-target activity as the basis for this effect. Propidium iodide cell cycle analysis demonstrated that treatment with either simvastatin or the related HMGCR antagonist atorvastatin significantly increased the sub-2N population and decreased the 2N population, indicative of cell death (Fig 3B). Atorvastatin impaired EBV-driven B-cell outgrowth (S4D Fig), even though it has not been shown to share the LFA-1 off-target effect.

HMGCR is the rate-limiting enzyme of the mevalonate pathway [37], which branches to produce either cholesterol or isoprenoids (Fig 3C). To determine if either or both branches were crucial for EBV-mediated primary B-cell outgrowth, we tested whether add-back of cholesterol precursor squalene (SQL) or isoprenoid precursor geranylgeranyl pyrophosphate (GGPP) could rescue outgrowth of simvastatin-treated, newly-infected B-cells. Interestingly, add back of either the HMGCR product mevalonate or the GGPS1 product GGPP nearly completely rescued EBV-driven B-cell outgrowth in the presence of simvastatin (Fig 3D), suggesting that isoprenoid synthesis and/or protein prenylation were critical. Cholesterol scavenging by EBV-upregulated LDLR (S2A and S2B Fig) may have supplied cells with sufficient cholesterol to unmask this GGPP requirement [64]. This result also suggests that simvastatin acts on-target to block B-cell outgrowth rather than by effects on LFA-1, which is not known to be geranylgeranylated.

To next investigate whether GGPP is important for growth or survival, newly infected primary B-cells and GM12878 LCL were treated with 2 μM simvastatin, in the absence or presence of GGPP rescue. Interestingly, simvastatin more strongly inhibited proliferation in newly infected cells but induced higher frequency of cell death in LCLs (Figs 3E, S4E and S4F). GGPP did not inactivate simvastatin, as RT-PCR analysis showed that cholesterol pathway targets and LDLR were upregulated by simvastatin blockade of HMGCR, even in the presence of GGPP (S4G Fig).

Substantial but incomplete GGPP rescue of LCL death may be accounted for by the prior observations that simvastatin off-target effects on LFA1 also cause LCL apoptosis [62]. Simvastatin induced cleavage of poly (ADP-ribose) polymerase (PARP), an event characteristic of apoptosis induction in newly infected cells, which could be mitigated with GGPP supplementation (Fig 3F), further supporting the notion of GGPP being a crucial metabolite that supports viral B-cell transformation.

### The Rab geranylgeranylation GGT-II complex is important for EBV-driven B-cell outgrowth

**Fig 4:**
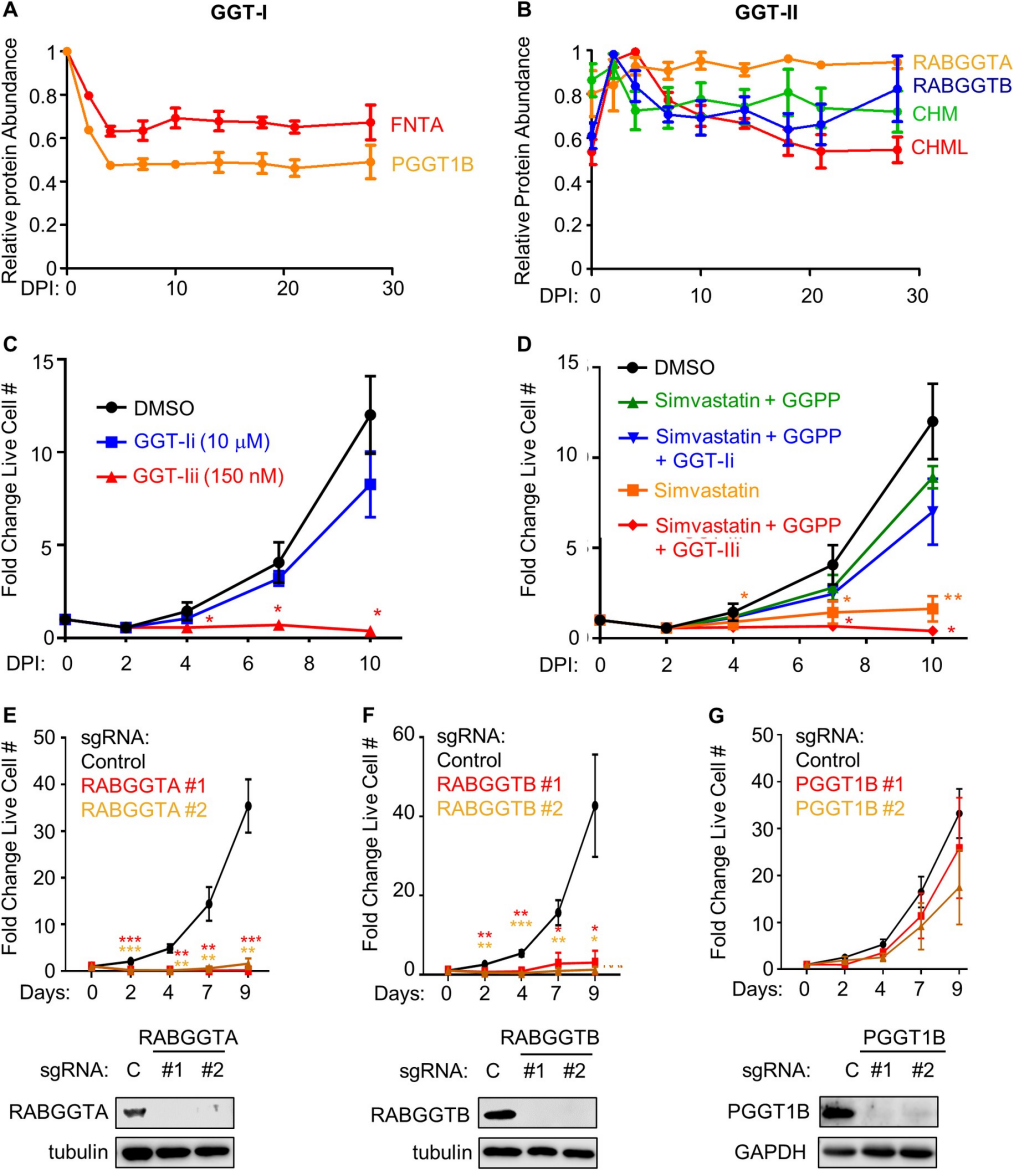
EBV induced GGT-II and its substrate GGPP are critical for EBV-infected primary human B-cell outgrowth. (A) Temporal traces of relative protein abundances for the GGT-I subunits FNTA and PGGT1B. Mean + SEM levels for three biological replicates are shown. (B) Temporal traces of relative protein abundances for GGT-II subunits RABGGTA, RABGGTB, CHM and CHML. Mean + SEM levels for three biological replicates are shown. (C) Growth curve analysis of newly infected primary B-cells cultured in the presence of DMSO, GGTI-2133 (GGT-Ii, 10 μM) or BMS-214662 (GGT-IIi, 150 nM) from 2–10 DPI. Inhibitors were refreshed at each timepoint. Mean + SEM fold change live cell numbers from n = 3 replicates are shown. *, p<0.05 (two-tailed t-test). (D) Chemical epistasis-growth curve experiments with newly infected primary B-cells treated as indicated. Cells were cultured with DMSO, simvastatin (2 μM), GGPP (2 μM), the GGT-I inhibitor GGTI-2133 at 10 μM (GGT-Ii) or the GGT-II inhibitor BMS-214662 at 150 nM (GGT-IIi) from 2–10 DPI. Simvastatin and GGPP were refreshed at each timepoint. Mean + SEM fold change live cell numbers from n = 3 replicates are shown. *, p<0.05; **, p<0.01 (two-tailed t-test). (E) Growth curve analysis of Cas9+ GM12878 LCL that express control (C) or independent *RABGGTA*-targeting sgRNAs, as indicated. Mean + SEM fold change live cell numbers from n = 4 replicates are shown. **, p<0.01; ***, p<0.005 (two-tailed t-test). Below, representative immunoblots (n = 2) of RABGGTA and tubulin load-control levels in WCL from LCLs expressing the indicated sgRNA are shown. (F) Growth curve analysis of Cas9+ GM12878 LCL that express control (C) or independent *RABGGTB*-targeting sgRNAs, as indicated. Mean + SEM fold change live cell numbers from n = 4 replicates are shown. *, p<0.05; **, p<0.01; ***, p<0.005 (two-tailed t-test). Below, representative immunoblot analysis (n = 2) of RABGGTB and tubulin load-control levels in WCL from LCLs expressing the indicated sgRNA are shown. (G) Growth curve analysis of Cas9+ GM12878 LCL that express control or independent *PGGT1B*-targeting sgRNAs are shown. Mean + SEM fold change live cell numbers from n = 4 replicates are shown. Below, representative immunoblot analysis (n = 2) of PGGT1B and GAPDH load-control levels in WCL from LCLs expressing the indicated sgRNA are shown.

Geranylgeranyltransferase (GGT) complexes transfer GGPP to small GTP-binding protein targets [65]. We asked whether GGT-I or GGT-II activity necessitates the GGPP dependency. GGT-I catalyzes GGPP addition to Ras superfamily proteins including Rac and Rho G proteins [66], whereas GGT-II mediates geranylgeranylation of Rab proteins to enable their membrane association [67]. Notably, EBV infection upregulated GGT-II but downmodulated GGT-I subunits (Fig 4A and 4B).

To test whether either GGT activity is critical for EBV-driven B-cell outgrowth, we performed chemical epistasis experiments with highly selective chemical antagonists. The GGT-II inhibitor BMS-214662 is 1000-fold selective for GGT-II over GGT-I, whereas the GGT-I inhibitor GGTI-2133 is reported to be 140-fold selective over GGT-II [63, 68, 69]. Dose-response experiments identified on-target GGT-I inhibitor activity on geranylgeranylation of the well-established target Rap1a/b [70] beginning at 2 μM, with full inhibition of Rap1a/b prenylation at 10 μM (S5A Fig). The GGT-II inhibitor was found to block prenylation of the GGT-II Rab protein target Rab13 at 150 nM. Notably, 150 nM GGT-II inhibitor also blocked phosphorylation of LMP2A target mTOR and induction of LMP1 target TRAF1 (S5B Fig).

At these biochemically determined EC50 concentrations, the GGT-II inhibitor but not the GGT-I inhibitor significantly impaired EBV-mediated primary B-cell outgrowth (Fig 4C). Similarly, an EC50 for inhibition of EBV-driven primary B-cell outgrowth was not determined for the GGT-I inhibitor, despite drug concentrations as high as 20 μM, whereas the GGT-II EC50 on primary B-cell outgrowth was found to be 80 nM (S5C and S5D Fig). Consistent with this conclusion, independent sgRNAs against the genes encoding GGT-II subunits RABGGTA and RABGGTB, but not against the GGT-I subunit PGGT1B, strongly decreased Cas9+ LCL growth and/or survival (Fig 4E–4G). Furthermore, sgRNAs targeting genes encoding GGT-II subunits were more strongly depleted from Cas9+ LCL pools after 21 days of growth than those targeting GGT-I subunits in our recent genome-wide CRISPR screen [71] (S5E–S5H Fig). RABGTTA or RABGTTB depletion by CRISPR editing reduced phosphorylation of LMP2A kinase targets SYK and PI3K subunit p85 (S6A and S6B Fig). Collectively, these results suggest that EBV upregulates GGPP production for Rab protein activation.

### EBNA3C-induced Rab13 is critical for LMP1/2A trafficking, LCL growth and survival

**Fig 5:**
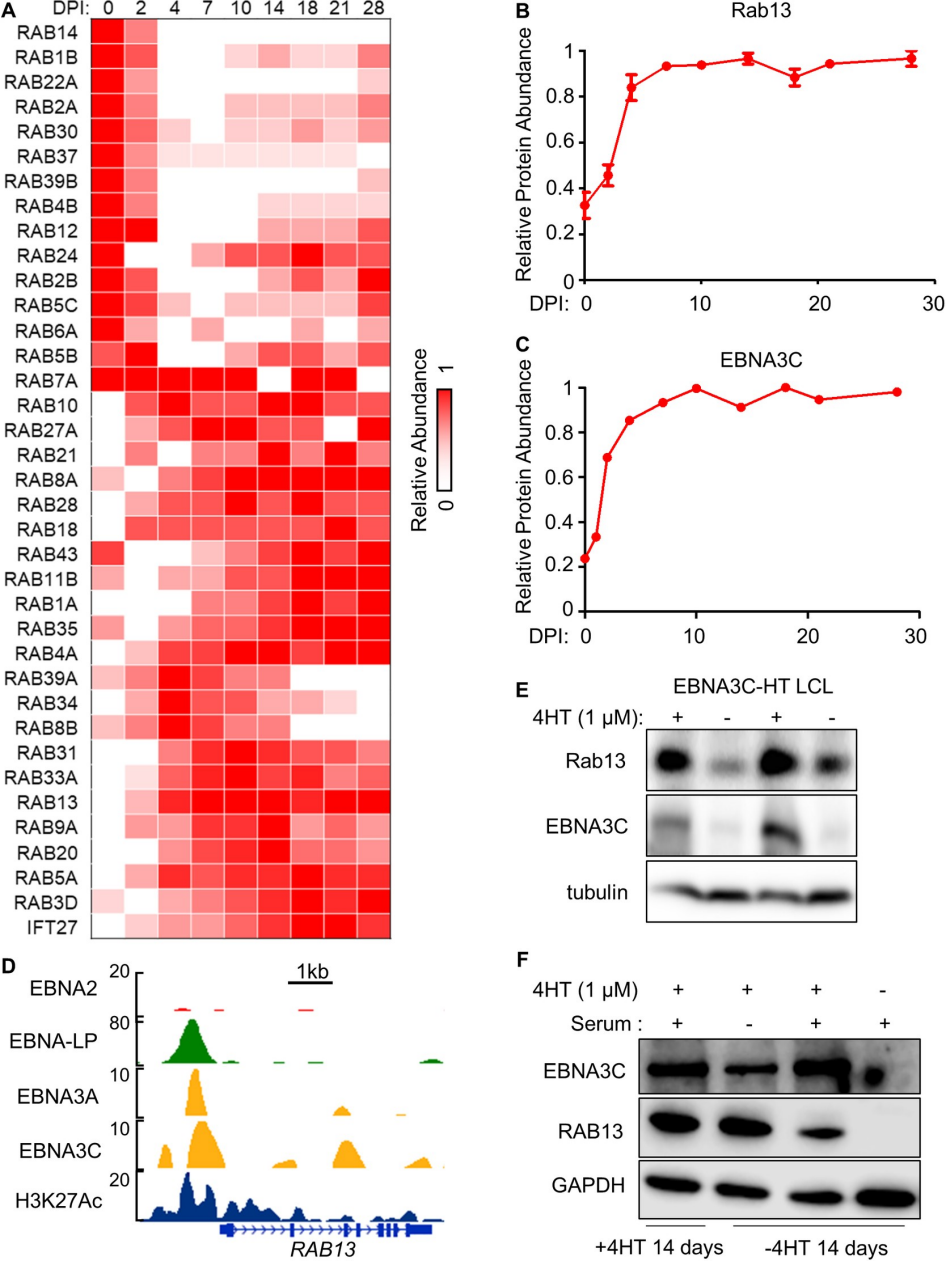
EBNA3C is important for EBV-mediated upregulation of Rab13 expression. (A) Heatmap representation of relative abundance profiles for all Rab family proteins detected in whole cell proteomic analysis of primary human B-cells infected by EBV at the indicated DPI, clustered by K-means analysis. Mean values from biological triplicate measurements for each Rab detected in the proteomic 28-day timecourse dataset were normalized to a maximum of one for each Rab. (B) Temporal traces of Rab13 relative protein abundance at the indicated DPI of primary human B-cell EBV infection. Mean + SEM levels for three biological replicates are shown. (C) Temporal traces of EBNA3C protein relative abundance at the indicated DPI of primary human B-cell EBV infection. (D) LCL ChIP-seq tracks for the indicated EBNAs or H3K27Ac epigenetic mark at the LCL *RAB13* locus. (E) Immunoblot analysis of conditional EBNA3C-HT LCLs grown in the presence of 4HT (1 μM) (EBNA3C permissive) or absence of 4HT (EBNA3C non-permissive) for 14 days. Representative blots of n = 5 experiments are shown. (F) Immunoblot analysis of conditional EBNA3C-HT LCLs grown in the presence of 4HT for 14 days (lane 1) or in the absence of 4HT for 14 days (lanes 2–4) and then treated with 4HT (1 μM) for 24 hours, where indicated. EBNA3C induction was done in media lacking 10% fetal bovine serum (serum) where indicated in order to suppress EBNA3C induction of cell cycle. See also S6C Fig.

We hypothesized that GGPP-modified Rab protein(s) important for EBV-mediated B-cell outgrowth would be upregulated upon primary B-cell infection. Of the 37 Rab proteins detected in our recent temporal proteomic analysis of EBV-mediated B-cell remodeling, 24 were upregulated at ≥ 1 time point (Fig 5A). Of these, cells expressing sgRNAs targeting *RAB13* were most strongly selected against in our 21 day LCL growth and survival screen [71]. We hypothesized that EBNA3C was important for *RAB13* induction by EBV, since Rab13 abundance was correlated with that of EBNA3C in newly infected B-cells (Fig 5B and 5C) and LCL ChIP-seq [23, 48, 49] demonstrated EBNA3C occupancy at the *RAB13* promoter. Indeed, conditional EBNA3C inactivation strongly downmodulated Rab13 expression in C19 LCLs, which express an EBNA3C/4HT-binding domain fusion protein [72] (Fig 5D). These results agree with prior microarray analyses, which found significant downmodulation of *RAB13* transcript upon C19 LCL EBNA3C inactivation [73]. Following EBNA3C conditional inactivation, add back of 4HT rapidly re-induced EBNA3C and Rab13 expression, even in the absence of serum, suggesting that EBNA3C effects on cell cycle do not underlie its ability to induce Rab13 (Figs 5F and S6C).

**Fig 6:**
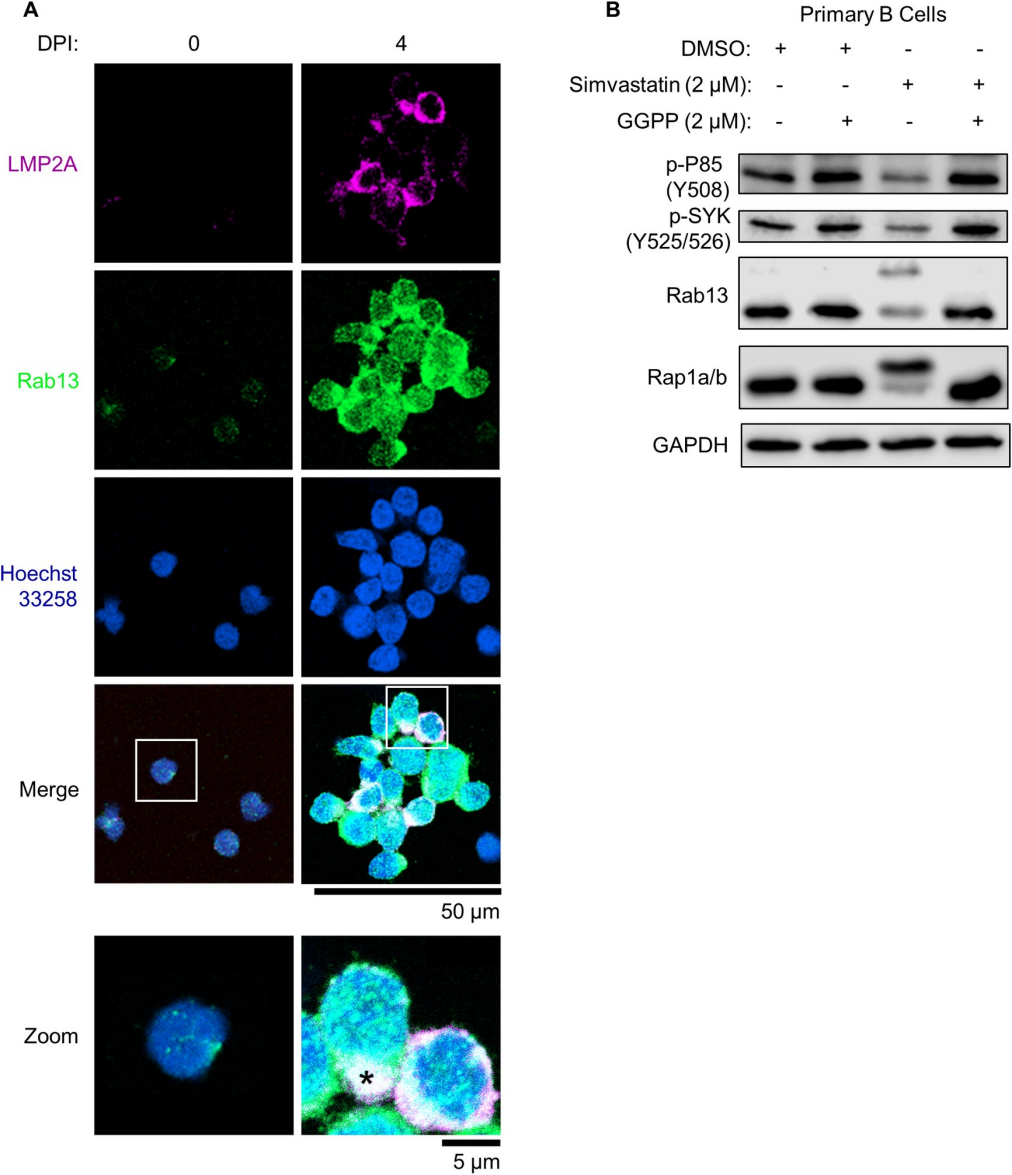
Rab13 colocalizes with LMP2A in newly infected primary B-cells. (A) Immunofluorescence micrographs of LMP2A, Rab13 and nuclear Hoechst 33528 uninfected versus infected primary human B-cells at 4 DPI. Merged images are shown with white boxes indicating inset images that have been further magnified. The asterisk denotes an area of strong Rab13 and LMP2A colocalization. Scale bars are indicated (50 μm for single-channel and merged images; 5 μm for inset images). See also S7A Fig. (B) Immunoblot analysis of WCL from newly infected primary B-cells cultured in the presence of DMSO, simvastatin (2 μM) or GGPP (2 μM) as indicated from days 2–7 post infection. Shown are representative blots from n = 3 experiments. P85 phospho-tyrosine 508 (Y508) and SYK phospho-tyrosines 525/526 (Y525/526) are markers of their activation, whereas unprenylated Rab13 and Rap1a/b migrate at higher electrophoretic mobilities. See also S8 and S9 Figs.

Immunofluorescence microscopy confirmed that Rab13 was upregulated by four days post EBV infection of primary B-cells (Fig 6A). At this timepoint, Rab13 and LMP2A colocalized in punctate regions, reminiscent of B-cell receptor (BCR) micro-clusters that form during antigen-dependent reactions (Figs 6A and S7A). We therefore hypothesized that Rab13 might be important for trafficking and activation of EBV latent membrane proteins, particularly in lymphoblastoid cells where LMP1 and 2A are highly expressed, co-localize and signal from lipid raft sites [74–78]. In support, we noted that three out of four *RAB13* targeting sgRNAs were strongly selected against in our 21-day CRISPR Achilles heel screen [71] (S7B Fig).

To further characterize potential Rab13 roles in support of LCL latent membrane protein function, we first tested whether simvastatin altered Rab13 prenylation and LMP signaling in newly infected B-cells. Interestingly, 2 μM simvastatin increased the electrophoretic mobility of Rab13 as well as Rap1a/b (Fig 6B), suggesting that they are each modified by prenylation in EBV-infected primary B-cells. Simvastatin downmodulated LMP2A-driven phosphorylation of the kinases SYK and PI3K subunit P85 and perturbed Rab13 and LMP2A trafficking in newly infected B-cells, resulting in diffuse staining patterns as opposed to focused distribution patterns DMSO vehicle treated cells (S8 Fig). Importantly, addition of GGPP to simvastatin treated cells rescued each of these phenotypes (Figs 6 and S8). Similarly, 150 nM GGT-II inhibitor treatment resulted in diffuse Rab13 and LMP2A staining patterns (S9 Fig), further implicating Rab13 geranylgeranylation as an important step in its ability to chaperone LMP2A.

**Fig 7:**
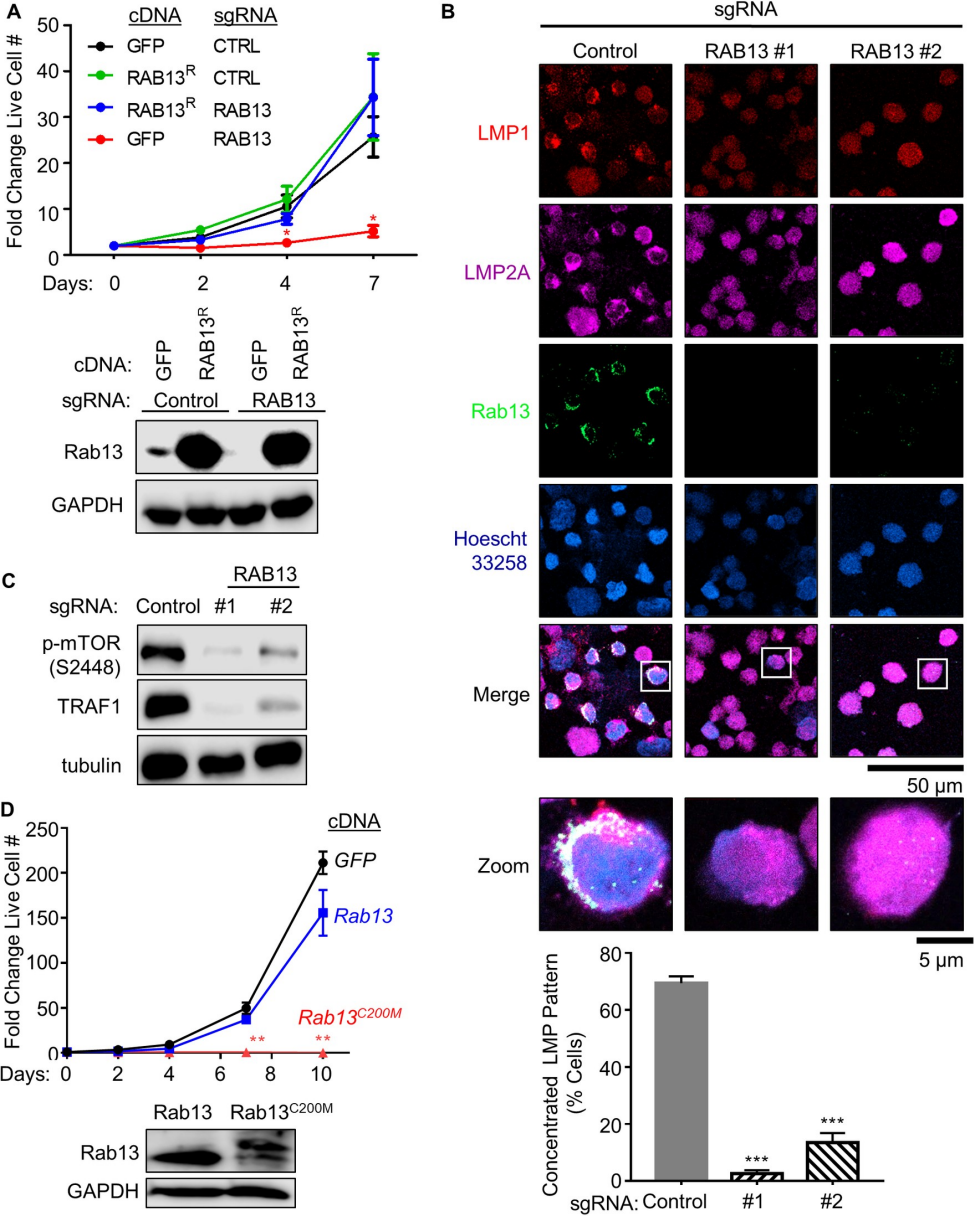
EBV-induced Rab13 is important for LCL LMP1 and 2A trafficking and signaling. (A) Growth curve analysis of Cas9+ GM12878 LCL that express either control GFP or rescue *RAB13* (RAB13^R^) cDNAs and either control or *RAB13*-targeting sgRNA. A silent mutation at the CRISPR PAM site abrogates Cas9 cleavage of the RAB13^R^ cDNA. Data show the mean + SEM fold change live cell values at the indicated timepoints for n = 3 replicates. *, p<0.05. Below, representative immunoblots from n = 3 replicates of WCL from LCLs expressing the indicated cDNA and sgRNA are shown. (B) Immunofluorescence micrographs of Cas9+ GM12878 LCL that express either control or *RAB13*-targeting sgRNAs, showing localization patterns of Rab13, LMP1 or LMP2A. Merged images are shown with white boxes indicating inset images that have been further magnified. Scale bars are indicated (50 μm for single-channel and merged images; 5 μm for inset images). Shown below are mean + SEM numbers of cells with concentrated LMP1 and 2A staining patterns in GM12878 expressing control versus RAB13 targeting sgRNAs, for which 200 cells were counted from n = 3 replicates. (C) Immunoblot analysis of WCL from Cas9+ GM12878 LCL that express either control or independent *RAB13*-targeting sgRNAs. Representative blots from n = 2 experiments are shown. mTOR phosphoSerine-2448 (S2448) is a marker of mTOR activation, whereas TRAF1 is an LMP1-induced LCL target gene. See also S10 Fig. (D) Growth curve analysis of GM12878 LCL that stably express either control GFP, wildtype Rab13 or a Rab13 point mutant in which the putative cystine 200 prenylation site has been changed to methionine to abrogate GGPP modification (Rab13^C200M^). Data show the mean + SEM fold change live cell values at the indicated timepoints for n = 3 replicates. Below, representative immunoblots from n = 3 replicates of WCL from LCLs expressing the indicated *RAB13* cDNA are shown.

We next tested whether Rab13 exerted a similar role in fully transformed LCLs. A Rab13-targeting single guide RNA (sgRNA) was used to functionally knockout Rab13 expression in Cas9+ GM12878 LCLs. Rab13 depletion markedly reduced LCL live cell numbers in a growth assay (Fig 7A). To validate that on-target effects on *RAB13* accounted for this phenotype, a *RAB13* allele, in which a silent point mutation at the protospacer adjacent motif (PAM) abrogated Cas9-mediated DNA editing, or control *GFP* cDNA were stably expressed in Cas9+ GM12878 LCLs. Effects of control versus sgRNA targeting endogenous *RAB13* were next tested in a 7-day outgrowth assay. Whereas *RAB13* sgRNA depleted endogenous Rab13 and strongly suppressed outgrowth of LCLs with control GFP cDNA expression, *RAB13* sgRNAs did not impair outgrowth of LCLs with rescue *RAB13* cDNA expression, confirming on-target CRISPR effects (Fig 7A).

We next investigated effects of *RAB13* targeting by independent sgRNAs on LCL LMP1 and 2A subcellular localization. Interestingly, Rab13 depletion by either sgRNA substantially altered LMP1 and LMP2A distribution: whereas LMP1 and LMP2A formed co-localized foci in LCLs with control sgRNA expression, as is typically observed in LCLs, they instead exhibited a diffuse pattern in LCLs with independent *RAB13* sgRNAs (Fig 7B). Consistent with prior reports that appropriate LMP trafficking is necessary for signaling from their cytoplasmic tail domains, Rab13 depletion diminished LMP1 induction of its well-characterized B-cell target TRAF1 (Figs 7C and S10A). Likewise, Rab13 loss strongly diminished LMP2A activation of mTOR, as evidenced by mTOR serine 2448 (S2448)-phosphorylation status (Fig 7C). Yet, Rab13 loss did not significantly change LMP1 or LMP2A abundance (S10B Fig), consistent with the hypothesis that Rab13 regulates LMP1/2A activity at the post-translational level.

**Fig 8:**
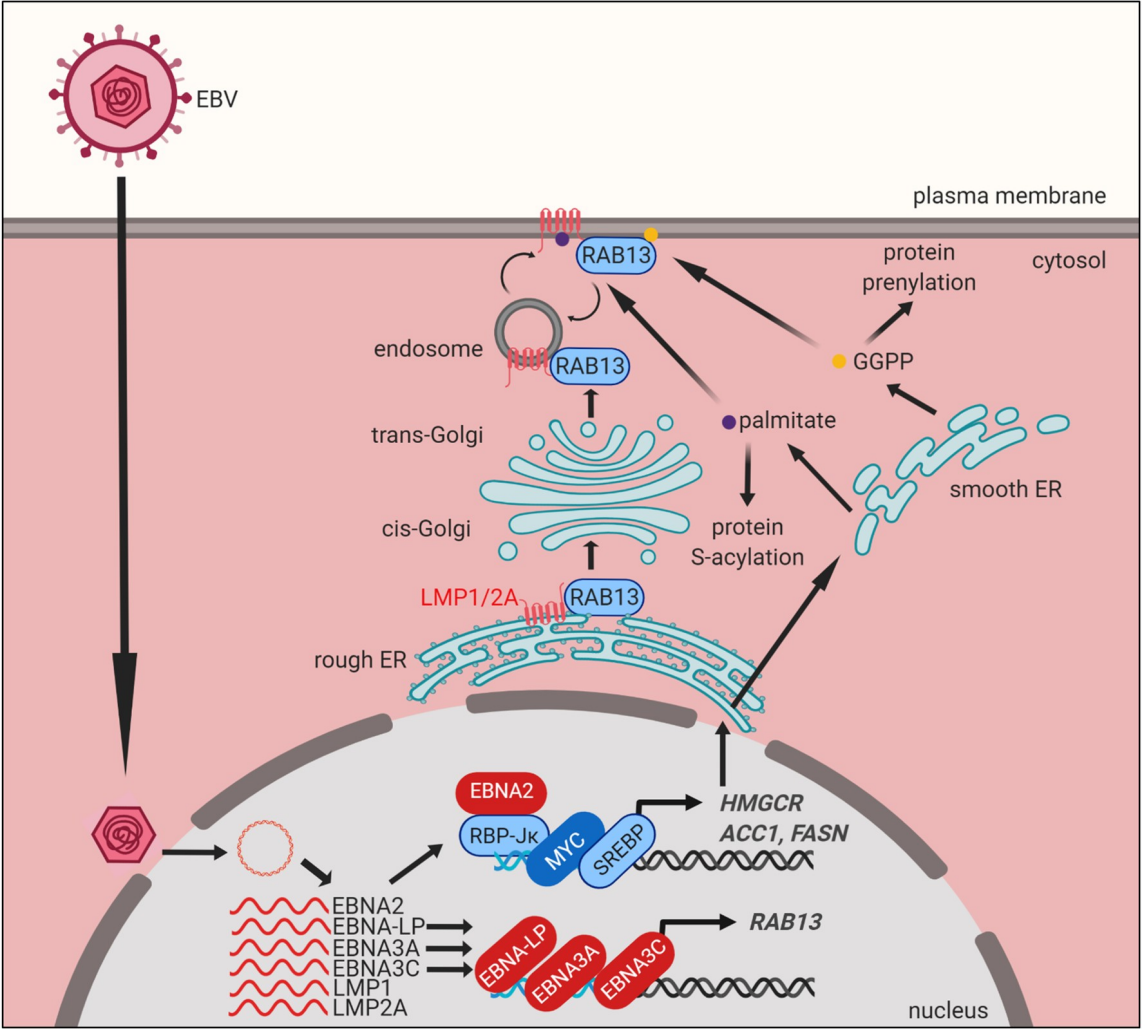
Model of EBV-induced cholesterol and lipid biosynthesis pathways remodeling and roles in LMP trafficking. EBV-encoded EBNA2 targets growth-promoting transcription factors such as MYC and SREBPs, which together with EBNA2 drive expression of key lipid biosynthesis genes. These metabolic programs induce mevalonate and fatty acid synthesis for B-cell remodeling and post-translational activation of target proteins. Acetyl-CoA-derived GGPP supports Rab geranylgeranylation and phospholipid synthesis. EBNA3C upregulates Rab13 expression, which then serves key chaperone roles in LMP1 and 2A trafficking and signaling.

Stable expression of a Rab13 point mutant, in which the putative prenylation site cysteine 200 was replaced by methionine (C200M), strongly impaired LCL growth and survival (Fig 7D), presumably via dominant-negative effects. The C200M mutation abolishes Rab13’s capacity for geranylgeranylation at this site, as the free thiol group required to react with geranylgeranyl pyrophosphate is absent on methionine. In agreement with the absence of geranylgeranylation, the Rab13 C200M mutant migrated more slowly than wildtype Rab13 on SDS-PAGE (Fig 7D). Notably, the C-terminal Rab13 amino acids SLG are removed after geranylgeranylation of cysteine 200, whereas the non-geranylgeranylated C200M variant retains its full-length sequence. Taken together, these observations indicate an important Rab13 role in LMP1 and LMP2A trafficking, likely to lipid raft signaling sites (Fig 8).

## Discussion

Diverse DNA viruses subvert host lipid biosynthesis pathways to support viral replication, including Kaposi’s sarcoma-associated herpesvirus (KSHV) [79, 80], human cytomegalovirus (HCMV) [81–83], herpes simplex virus (HSV) [84, 85] and vaccinia virus [86]. Interestingly, KSHV subverts hypoxia inducible factors (HIF) to induce metabolic remodeling in B-cells [87], whereas in epithelial cells it targets glutamine and asparagine metabolic pathways, while suppressing glycolysis, to support transformed cell growth [88, 89]. Yet, comparatively less is known about how EBV remodels lymphocyte metabolic pathways to support cell transformation, growth and survival. While EBV strongly upregulates aerobic glycolysis in newly infected B-cells [25], presumably to supply building blocks for anabolic metabolism pathways, key downstream lipid biosynthesis pathways upregulated by latent EBV infection have remained to be identified.

The studies presented here highlight EBV-induced mevalonate, GGPP, fatty acid biosynthetic and downstream Rab13 pathways as being critical for newly infected B-cell outgrowth (Fig 8). EBNA2 and its targets MYC and SREBP2 were found to play major roles in remodeling of these key pathways in early B-cell transformation. Further studies are required to fully define how these host and viral transcription factors may function together on DNA to induce key lipogenic target genes. SREBPs interact with MYC at promoters of stemness-controlling genes [90, 91], and LCL ChIP-seq identifies that they co-occupy promoters of LCL cholesterol and lipid metabolism pathway genes, together with EBNA2. Further suggesting interdependent roles, EBNA2, MYC and the SCAP/SREBP axis were found to cross-regulate one another’s abundance in LCLs. We speculate that EBNA2 and SREBPs may initially induce cholesterol and lipid pathway target genes, and that this activity is then amplified by MYC.

LMP1 has been reported to activate lipogenesis via mTOR and SREBP1 in nasopharyngeal carcinoma (NPC) cells, which typically express the latency II program [92]. However, our studies instead point to important EBNA roles in lipid pathway remodeling in newly infected B-cells. First, newly infected cells do not have high levels of LMP1 over the first several days post-infection when key targets such as HMGCR and ACC1 are initially upregulated. Second, LCL ChIP-seq demonstrates only modest NF-κB subunit occupancy at the *HMGCR* and *ACACA* promoters. Instead, EBNA2, MYC and SREBPs highly co-occupy these key promoters. LMP2A may nonetheless have critical roles in maintaining cholesterogenic and lipogenic programs at later timepoints by stimulating PI3K/AKT/mTOR flux [93].

We initially suspected that EBV upregulates mevalonate synthesis to supply cholesterol for lipid raft and membrane synthesis in support of B-cell lymphoblast transformation. However, using a chemical epistasis approach [63], we unexpectedly found that simvastatin impaired EBV-infected primary B-cell outgrowth by preventing GGPP synthesis and Rab protein activation. While off-target simvastatin effects on LFA-1 and on LMP1 dissociation from lipid rafts have previously been reported in LCLs [62], simvastatin effects on EBV primary B-cell outgrowth and survival could be nearly completely rescued by GGPP supplementation. In further support of on-target statin activity underlying effects on EBV-driven B-cell outgrowth, a chemically distinct statin, atorvastatin, similarly blocked EBV-infected B-cell outgrowth. Lower LMP1 and LMP1-induced LFA-1 expression in newly infected B-cells [29] may have reduced the impact of the previously reported HMGCR-independent effects of simvastatin [62] in our experiments. Cholesterol also plays important roles in LMP1 and LMP2A trafficking [62, 75, 94, 95], and the mevalonate pathway may therefore also support this key aspect of EBV oncoprotein function in microenvironments with limited extracellular LDL.

While to our knowledge GGPP has not previously been studied in EBV-infected cells, key mevalonate pathway and GGPP roles have been identified in other B-cell contexts. Importantly, CD40-mediated B cell activation upregulates the mevalonate pathway in primary B-cells, where GGPP supports antigen-presenting cell function and T-cell activation by mediating CD80, CD86 and HLA-DR upregulation [96]. However, specific GGPP targets, such as Rab proteins that may function in trafficking of CD40 or of T-cell stimulatory molecules, remain to be identified. Given how closely LMP1 mimics CD40, we hypothesize that LMP1 may reinforce EBNA2-driven mevalonate metabolism and GGPP upregulation in the lymphoblastoid phase of B-cell transformation. Additional B-cell GGPP roles appear likely, since geranylgeranylation supports proliferative capacity and survival of multiple EBV-negative lymphomas and multiple myeloma B-cells in culture [97–100].

Our results highlight the interplay between Rab13, LMP1 and LMP2A in B-cell transformation and in transformed cell growth and survival. Rab13 has pleiotropic roles, including the regulation of glucose transporter 4 trafficking [101], macrophage phagocytosis [102, 103], autophagy [104], lymphocyte trafficking [105], anoikis and metastasis [106–108]. Notably, LMP1 traffics to intracellular membranes to initiate signaling or to be secreted in exosomes [75, 94, 95, 109–112], though the chaperone protein(s) required for intracellular sorting are incompletely defined. Our results suggest that EBV co-opts Rab13 for yet another function: to chaperone oncogenic LMP1 and LMP2A to appropriate sub-cellular compartments where they can then activate downstream pathways. Rab13+ punctate structures observed in newly infected cells may represent unprenylated, inactive Rab13 pools that are subsequently activated by guanine exchange factors at the cell membrane [113].

Given that LMP1 and LMP2A can signal from intracellular membranes [77, 111, 114, 115], it is possible that Rab13 functions to directly promote LMP movement to plasma membrane and endosomal compartments. Alternatively, Rab13 may undergo cycles of activation and inactivation to facilitate LMP recycling between the plasma membrane and endosomes, in a manner similar to that with occludin in epithelial cells [116]. Notably, the prenylated Rab acceptor 1 (PRA1/RABAC1) associates with LMP1 at the Golgi complex and prevents LMP1 retrograde translocation back to the ER [110]. PRA1 interacts with multiple prenylated Rab GTPases [117, 118] and facilitates their delivery to cell membranes by counteracting the guanine nucleotide dissociation inhibitor GDI, a protein that actively solubilizes membrane-bound Rab proteins [119]. We speculate that LMP1 association with PRA1 may require Rab13 as an intermediary binding partner.

The crucial EBNA3C role in support of LCL Rab13 expression raises the interesting question of how LMP1 and LMP2A traffic and signal in cells with latency II expression where EBNA3C is not expressed, such as in germinal center B-cells, Hodgkin Reed Sternberg (RS) or nasopharyngeal carcinoma tumor cells [12, 31, 32, 77, 120]. Interestingly, RNA profiling datasets from classic Hodgkin’s lymphoma, the HL subtype most closely associated with EBV, identify RS cell *RAB13* mRNA expression [121]. Since germinal center B-cells are the RS cell of origin and support EBV latency II expression, alternative mechanisms for Rab13 induction may therefore be operative in this important B-cell developmental state. Alternatively, other Rab protein(s) may act redundantly with Rab13 to support LMP1 and LMP2A transport in B and epithelial cells with EBV latency II expression. For instance, LMP1 was recently found to upregulate Rab11, which can have major roles in protein trafficking [122].

These studies identify multiple EBV-induced metabolic pathway enzymes that may serve as attractive therapeutic targets to prevent or halt EBV-driven lymphoproliferative disease, which could perhaps be targeted by available small-molecule inhibitors as prophylaxis during periods of profound immunosuppression. These could potentially include key druggable enzymes in the mevalonate, geranylgeranylation or fatty acid biosynthetic pathways. Of particular interest are simvastatin and atorvastatin, since the concentrations of these widely used statins at which we observed inhibition of EBV-mediated B-cell outgrowth *in vitro* can likewise be achieved in humans in vivo [123]. Simvastatin multi-target effects on LFA-1 and LMP1 lipid raft dissociation [62] further underscore its potential in counteracting EBV transformed lymphoblastoid B-cell growth. It will be of significant interest to test statin targets identified herein with recently developed humanized mouse models of EBV lymphoproliferative disease [124].

## Materials and methods

### Culture of cell lines

293T were purchased from American Type Culture Collection (ATCC) and cultured in DMEM with 10% fetal calf serum (FCS, Gibco). GM12878 lymphoblastoid cells were obtained from Coriell. GM12878 Cas9+ cell lines were previously described [71]. The 2-2-3 EBNA2HT LCL with conditional EBNA2 allele was a kind gift from Bo Zhao and Elliott Kieff (Brigham & Women’s Hospital, Harvard Medical School). 2-2-3 LCLs express EBNA2 fused to a modified estrogen receptor 4HT-binding domain. In the presence of 4HT, the EBNA2HT allele localizes to the nucleus and is active, but upon 4HT withdrawal is redistributed to the cytosol where it is destabilized. 2-2-3 LCLs were maintained in the presence of 1 μM 4-hydroxytamoxifen (4HT). To remove 4HT, cells were washed five times with 4HT-free media, including two incubations for 30 minutes, and then re-seeded at 100,000 cells per mL in media with or without 4HT, as indicated. Cells were then grown for 48 hours and harvested for RNA extraction and cell lysate preparation. The C19 EBNA3CHT was a kind gift from Bo Zhao and Elliott Kieff. It was maintained in the presence of 1 μM 4HT. To remove 4HT, cells were grown in 4HT-free media for three days, washed five times as described above, and re-seeded at 100,000 cells per mL. Cells were then grown for 14 days before harvesting for RNA extraction and cell lysate preparation. The conditional P493-6 LCL was a kind gift from Micah Luftig (Duke University). P493-6 cells have a conditional EBNA2HT allele similar to 2-2-3 LCLs, and also have a TET-Off exogenous *MYC* allele. In the absence of tetracyclines, high level exogenous *MYC* is induced. P493-6 cells were maintained continuously in the absence of 4HT or doxycycline to induce a BL-like state of high MYC expression. To grow in the lymphoblastoid cell state (intermediate MYC), P493-6 cells were grown in the presence of both 1 μM 4HT and doxycycline. To induce a low MYC state and consequently G1 growth arrest, cells were grown in the absence of 4HT and presence of 1 μM doxycycline. After 48 hours of growth in any of these conditions, cells were collected for lysate preparation. For selection, 200 μg/mL hygromycin (Calbiochem) or 3 μg/ml puromycin (Invitrogen) was used. B95-8 cells and P3HR-1 cells with conditional ZTA-HT alleles responsive to 4HT were kind gifts from Eric Johannsen and Elliott Kieff. All B-cells were cultured in RPMI-1640 (Invitrogen) supplemented with 10% standard FBS and penicillin-streptomycin in a humidified incubator at 37ᵒC and at 5% CO_2_. All cells were routinely confirmed to be mycoplasma-negative by Lonza MycoAlert assay (Lonza).

### Ethics statement

Discarded, de-identified leukocyte fractions left over from platelet donations collected from volunteer donors following institutional guidelines were obtained from the Brigham and Women’s Hospital Blood Bank. Our studies on primary human blood cells were approved by the Brigham & Women’s Hospital Institutional Review Board. All samples were anonymized.

### Primary human B-cell isolation and culture

Platelet-depleted venous blood obtained from the Dana-Farber Cancer Institute blood bank were used for primary human B cell isolation, following our Institutional Review Board-approved protocol for discarded and de-identified samples. RosetteSep and EasySep negative isolation kits (STEMCELL Technologies) were used sequentially to isolate CD19+ B-cells with the following modifications made to the manufacturer’s protocols. For RosetteSep, 40 μL of antibody cocktail was added per mL of blood and then layered onto Lymphoprep density medium for centrifugation. For EasySep, 10 μL of antibody cocktail was added per mL of B cells, followed by 15 μL of magnetic bead suspension per mL of B cells. After negative selection, the cells obtained were routinely ≥95% positive for CD19, a nearly pan-B cell surface marker (though not strongly expressed on plasma cells). For most experiments, cells were cultured in RPMI-1640 (Invitrogen) supplemented with 10% standard FBS and penicillin-streptomycin. Cells were cultured in a humidified incubator at 37ᵒC and at 5% CO_2_.

### EBV infection of primary B-cells

EBV B95-8 virus was produced from B95-8 cells with conditional ZTA expression and purified by filtration through a 0.45 μm filter [125, 126]. EBV titer was determined experimentally by transformation assay. The P3HR-1 EBV strain was produced from P3HR-1 cells with conditional ZHT expression [125, 127]. Genomic DNA content of preparations of this non-transforming virus were quantitated by PCR for *BALF5* on total DNA extracted, and cross-compared with levels from B95-8 virus preparations. The plasmid pHAGE-BALF5 was used for generation of standard curves of EBV genome copy number. Calculated genome copy numbers were used to normalize B95-8 and P3HR-1 input DNA amounts. UV irradiation of B95-8 virus supernatants was performed at a cumulative intensity of 3J per square centimeter on ice, to prevent heat-induced virus degradation. EBV was added to purified B-cells at an MOI of 0.1 (250 μL of supernatant from ZHT cells 5 days after ZHT stimulation, with washout of 4HT after 24 hours, per million purified B-cells). We also ensured equal infection by performing quantitative PCR on intracellular DNA extracted from cells and by anti-EBNA1 (OT1x) immunofluorescence microscopy at 24 hours post-infection following at least five washes. Cells were cultured in a humidified chamber at 37˚C in RPMI/10% FCS. For most experiments, virus-containing media was replaced with virus-free media for subsequent experimentation two days post-infection.

### Antibodies and reagents

Antibodies against the following proteins were used in this study: ACC1 (Proteintech, Cat#21923-1-AP), HMGCR (Proteintech, Cat#13533-1-AP), SCAP (Proteintech, Cat#12266-1-AP), DDX1 (Bethyl, Cat#A300-521A), DDX46 (Proteintech, Cat#16927-1-AP), phospho-PI 3-kinase p85α (Tyr 508) (Santa Cruz, Cat#12929), phospho-Syk (Tyr525/526) (Cell Signaling, Cat#2710), RPA1A/B (R&D Systems, Cat#AF3767-SP), PGGT1B (Santa Cruz, Cat#376655), MYC (Santa Cruz, Cat#764), EBNA2 (mouse monoclonal antibody clone PE2, kindly provided by Dr Jeffrey Cohen), EBNA3C (Abcam, Cat#16128), RABGGTA (Proteintech, Cat#14448-1-AP), RABGGTB (Bethyl, A304-323A), Rab13 (Santa Cruz, Cat#517224), phospho-mTOR (Ser2448) (Cell Signaling, Cat#2971), TRAF1 (Cell Signaling, Cat#4715), GAPDH (Abcam, Cat#8245), α-tubulin (Abcam, Cat#7291), horse anti-mouse IgG HRP-linked antibody (Cell Signaling, Cat#7076), goat anti-rabbit IgG HRP-linked antibody (Cell Signaling, Cat#7074), goat anti-rat IgG HRP-linked antibody (Cell Signaling, Cat#7077), Alexa Fluor 488 goat anti-mouse (ThermoFisher, Cat#A-11029), Alexa Fluor 594 goat anti-rabbit (ThermoFisher, Cat#NC0414256), Alexa Fluor 647 goat anti-rat (ThermoFisher, Cat#A-21247), LMP1 (Abcam, Cat#136633), LMP2A (rat monoclonal antibody clone 4E11, kindly provided by Richard Longnecker).

The following chemicals were obtained from Sigma-Aldrich: fatostatin hydrobromide (Cat#F8932), simvastatin (Cat#S6196), atorvastatin calcium salt trihydrate (Cat#PZ0001), geranylgeranyl pyrophosphate (GGPP) (Cat#G6025), squalene (Cat#S3626), Mevalonate acid lithium salt (Cat #50838), GGTI-2133 (Cat#G5294), BMS-214662 hydrochloride (Cat#BM0008), doxycycline hyclate (Cat#D9891), (Z)-4-hydroxytamoxifen (Cat#H7904). Hoechst 33258 (Cat#H21491) and propidium iodide (Cat#P3566) were obtained from ThermoFisher Scientific.

### Immunoblot analysis

Cells were lysed in a modified TNE buffer (50 mM Tris-HCl pH 7.5, 150 mM NaCl, 1 mM EDTA, 0.5% NP-40, 0.5% sodium deoxycholate, 0.1% SDS) supplemented with protease inhibitor cocktail (Roche cOmplete Mini EDTA-free Protease Inhibitor Cocktail, Cat# 4693159001), 2 mM sodium pyrophosphate, 10 mM β-glycerophosphate and 1 mM PMSF on ice for 15 minutes. The crude cell lysate was then sonicated on ice for 5 minutes using a probe sonicator at the maximum setting and centrifuged at 17,000 x g for 15 minutes. The supernatant was mixed with 5X Laemmli loading buffer and boiled for 10 minutes. Samples were loaded onto and electrophoretically resolved on 12% or 4–20% gradient gels and transferred onto nitrocellulose membranes for 45 minutes at 100 V at 4˚C. Membranes were probed with primary antibodies, typically at a 1:1000 dilution overnight at 4˚C and with HRP-conjugated secondary antibodies in 5% skim milk/PBST for 1 hour. Blots were imaged on Carestream or Li-Cor workstations.

### Flow cytometry

Cells were washed once with cold PBS supplemented with 0.5% bovine serum albumin (BSA). Cells were then incubated with a 1:100 dilution of fluorophore-conjugated primary antibody (and, if applicable, a 1:500 dilution of fluorophore-conjugated secondary antibody) in PBS with 0.5% BSA for 1 hour. Cells were pelleted and resuspended in 400 μL of PBS, strained into flow cytometry-compatible tubes and processed immediately. Sample processing was performed either immediately or within 24 hours after staining. Flow cytometric data was acquired with a BD FACSCalibur instrument and analysis was performed with FlowJo. For propidium iodide cell cycle analysis, samples were collected over indicated time points and fixed in 70% ethanol overnight. For cell cycle analysis, fixed cells were treated with Staining Buffer (Propidium Iodide, 5μg/ml; RNase A, 40μg/ml; 0.1% Triton X-100; PBS) for 30 minutes at room temperature and analyzed by FACS. The FACS data were further analyzed with FlowJo V10.

### Immunofluorescence

Cells were seeded on glass slide and fixed with 4% PFA solution for 10 minutes. Fixed cells were permeabilized with 0.5% Triton X-100/PBS solution and blocked with 20% newborn goat serum (NGS). Subsequently, cells were incubated with a cocktail of primary antibodies against LMPs and Rab13 for an hour and then a cocktail of secondary antibodies for an additional half hour. Finally, cells were incubated with a solution of Hoechst 33258 (10 μg/mL) for 10 minutes to stain nuclear DNA and dehydrated sequentially from 70% to 90% to 100% ethanol. ProLong anti-fade was applied to the slide and sealed with a No. 1.5 coverslip. Image acquisition was performed at the Brigham and Women’s Hospital core facility with the Zeiss LSM 800 instrument. Image analysis was performed with the Zeiss ZEN Lite (Blue) software. For measurements of cell diameter, differential interference contrast (DIC) microscopy was performed and cells were picked out by eye from the micrographs. Circular ROIs were drawn around the cells and diameters were automatically computed by the ZEN Lite (Blue) software.

### Growth curve analysis

Newly infected primary human B-cells were seeded at 500,000 per well in a volume of 1 mL and grown for two days before measurements of cell densities were made. For GM12878 lymphoblastoid cells, 100,000 cells were seeded per well in a volume of 1mL and grown for two days prior to measurements of cell densities. For cell density measurements, cells were pelleted and resuspended in the same volume of media and counted with the TC20 automatic cell counter (Bio-Rad). After measurements had been taken, cells were passaged accordingly to give 100,000 cells per mL and grown for two more days. The same procedure was repeated to obtain measurements at subsequent timepoints. At each time point, after passaging, cells were treated with the appropriate inhibitor and/or rescue metabolite(s) at the indicated concentrations. Growth was then calculated using the initial seeding density for normalization.

### CRISPR editing in GM12878 LCL

Single guide RNA (sgRNA) constructs were generated as previously described [128] using sgRNA sequences from the Broad Institute Avana or Brunello Libraries. Sequences used to construct the sgRNA-encoding plasmids can be found in S3 Table. LCL CRISPR editing was performed as previously described [128]. Briefly, lentiviruses encoding sgRNAs were generated by transient transfection of 293T cells with packaging plasmids and pLentiGuide-Puro plasmids. GM12878 cells stably expressing Cas9 were transduced with the lentiviruses and selected with 3 μg/mL puromycin for three days before replacement with antibiotic-free media. For rescue experiments or cDNA overexpression, 293T cells were transiently transfected to produce lentiviruses that carry the rescue/overexpression cDNA and a hygromycin resistance marker. GM12878 cells were transduced with rescue lentiviruses and selected with 200 μg/mL hygromycin for at least one week before transduction with sgRNA-encoding lentiviruses. CRISPR editing and rescue cDNA expression were confirmed by immunoblotting. To construct the rescue *RAB13* (RAB13^R^) vector, we made use of the Genscript GenParts synthesis service to synthesize a cDNA fragment containing wild-type *RAB13* with cytosine 141 mutated to thymidine (c141t) and guanosine 162 mutated to thymidine (g162t), as well as flanking attB1 and attB2 sites. Engineering the c141t and g162t mutations at the PAM sites confers resistance to Cas9-mediated cutting by sgRNA #1 and sgRNA #2, respectively. We also introduced a stop codon at the end of the coding sequence. We performed BP cloning of the gene fragment into a donor vector, pDONR223. The resultant entry vector, pDONR223-RAB13^R^, was used for LR cloning with pLX_TRC313, a destination vector which carries a hygromycin resistance marker. Because pLX_TRC313 has a C-terminal V5 epitope tag sequence which could interfere with Rab13 function, the engineered cDNA fragment used for BP cloning contained a stop codon at the end of the coding sequence but upstream of the 3’ attB sequence. The expression vector for RAB13 C200M point mutation expression performed as follows. A Gateway entry clone encoding sgRNA-resistant *RAB13* with C200 residue mutated to methionine was generated using GenScript’s GenPart synthesis service. Specifically, the codon encoding cysteine at position 200 (TGC on its DNA equivalent) was mutated to encode methionine (ATG) at the same position. The C200M mutation abolishes the capacity for Rab13 geranylgeranylation at this site as the free thiol group required to react with geranylgeranyl pyrophosphate is absent on methionine. Although a number of earlier studies [113, 129] made use of truncation mutants lacking the last four amino acids (CSLG) to render Rab13 geranylgeranylation-incompetent, we decided to refine the mutation strategy with a single amino acid change to test more precisely the requirement of C200 for geranylgeranylation. Subsequently, we performed Gateway cloning to generate a retroviral Rab13^C200M^ stable expression construct with a puromycinc resistance marker using vector MSCV-N-FLAG-HA-IRES-PURO (Addgene plasmid # 41033).

### Quantitative PCR

Reverse transcription-quantitative PCR analysis of mRNA abundance was performed on a BioRad CFX Connect Real-time PCR detection system, using Power SYBR Green RNA-to-CT 1-Step Kit (Applied Biosystems) for 40 cycles. Expression values relative to 18S rRNA or GAPDH expression were calculated using CFX Manager Software. Quantitative PCR of viral genome copies utilized host cell GAPDH gene copy number as control. Primer sequences can be found in S4 Table.

### Proteomic analysis

Full details of how multiplexed tandem mass tag-based proteomic data was obtained were recently reported [26]. Briefly, primary human B cells were isolated from platelet-depleted venous blood following our Institutional Review Board-approved protocol for discarded and de-identified samples. RosetteSep and EasySep negative isolation kits (STEMCELL Technologies) were used sequentially to isolate CD19+ B-cells, which were always ≥95% positive for CD19. For each of three biological replicates, B-cells were isolated from four anonymous human donors. Uninfected B-cells were stained with anti-CD19 antibody and propidium iodide and a FACSort was performed for live CD19+ B-cells, in order to control for the effects of FACS at subsequent timepoints. B95-8 strain EBV was added to the remaining purified B-cells at a multiplicity of infection of 0.1. Cells were cultured in RPMI-1640 (Invitrogen) supplemented with 10% standard FBS and penicillin-streptomycin in a humidified incubator at 37ᵒC and 5% CO2, maintaining cultures from each B-cell donor in separate flasks. Upon reaching each requisite time point, cells from all four donors were stained with antibody against CD23, a surrogate marker of EBV-infected cells upregulated by EBNA2 early after EBV infection. Live CD23+ cells were sorted on the BD FACSAria cytometer. Samples from each donor were sorted sequentially. Immediately following the sort, cells were lysed and whole cell lysates (WCL) and plasma membrane (PM) samples prepared. Samples were combined at constant ratios at the cell lysis step. The three biological replicate time courses were each performed at >1 month apart. Subsequent downstream processing was performed as described [26].

Pathway Analysis was performed using the Database for Annotation, Visualization and Integrated Discovery (DAVID) [130] version 6.8 with default settings. A given cluster was always searched against a background of all proteins quantified within the relevant experiment. Proteins were included in this analysis if they were (1) contained in the metabolism gene list published by Birsoy and colleagues [36] and (2) quantified in all three experiments. The cluster that demonstrated >2-fold upregulation at 4 days post-infection with a p-value cut-off of 0.075 was searched against the full list to determine enriched terms. The mass spectrometry proteomics data used for this paper have been deposited to the ProteomeXchange Consortium (http://www.proteomexchange.org/) via the PRIDE partner repository and can be retrieved with the dataset identifier PXD013034.

#### ChIP-seq analysis

ChIP-seq raw data was obtained from published LCL datasets for EBNA2 [17] (GEO Accession number GSE29498, performed in IB4 LCLs with anti-ENBA2 antibody PE2), EBNA-LP [131] (GEO GSE49338, performed in IB4 LCLs with anti-EBNA-LP antibody JF186) EBNA3A [22] (GEO GSE59181, performed in LCLs [132] reported in which recombinant EBV encoded an HA-epitope tagged EBNA3A; HA was used for ChIP-seq), EBNA3C [24] (GEO GSE52632, performed in LCLs reported [132] in which recombinant EBV encoded an HA-epitope tagged EBNA3C; HA was used for ChIP-seq). GM12878 LCL ChIP-seq data for factors other than EBNA were downloaded from the ENCODE consortium [23, 49]. ChIP-seq reads were mapped onto hg19 with bowtie with the following parameters: -k 1 -m 1 [133]. Duplicates were removed from mapped reads with Picard (https://github.com/broadinstitute/picard) and uploaded onto a custom session of the WashU Genome Browser as previously described [23, 134]. ENCODE ChIP-seq tracks for GM12878 were loaded onto the WashU Genome Browser session for visualization [49].

### K-means clustering for Rab Protein Expression analysis

Data for expression of Rab proteins was extracted from the proteomic analysis of EBV-driven primary human B-cell outgrowth [26]. For each protein in each of three biological replicates, data was normalized to a maximum of one, then averaged and further normalized to a maximum of one. k-means clustering was then performed using Cluster 3.0 (Stanford University) with 100 iterations and visualized using Microsoft Excel.

## Supporting information

S1 Fig Temporal flow cytometry analysis of EBV-induced remodeling in primary B-cell size. (A) FACS forward scatter cell size measurements of live CD19+ primary human B-cells at the indicated days post-infection (DPI) by B95-8 EBV at a MOI of 0.1. Shown is a representative experiment of n = 3 replicates. Of note, cells were not gated for CD23 as a marker of EBV infection. (B) Propidium iodide (PI) cell cycle and forward scatter cell size FACS analysis are shown for the indicated DPI timepoints from the experiment shown in panel S1A. Data were representative of n = 3 experiments. (C) Propidium iodide (PI) cell cycle analysis for the indicated DPI timepoints from the experiment shown in panels S1A-B. Data were representative of n = 3 experiments. (D) Mean +SEM values of the numbers of cell identified as G1 or as in either G2 or S phases (G2+S) of the cell cycle by the PI analysis at the indicated DPI timepoints from n = 3 replicates, including the experiment presented in panels S1A-C. *, p<0.05; **, p<0.01. (two-tailed t-test).
(TIF)

S2 Fig EBV upregulates the LDL receptor in newly infected primary human B-cells. (A) Temporal traces of whole cell LDL receptor (LDLR) relative protein abundances at the indicated DPI of primary human B-cell EBV infection. Data show the mean + SEM of n = 3 biological replicates. (B) Temporal traces of plasma membrane (PM) LDLR relative protein abundances at the indicated DPI of primary human B-cell EBV infection. (C) Schematic diagram showing *de novo* lipid synthesis pathway conversion of glucose-derived acetyl-CoA into end products. NADPH-dependent acetyl-CoA reduction produces palmitate, which can be directed to one of three routes: (1) oxidation via the fatty acid β-oxidation pathway to produce reducing power in the form of NADH and ultimately, ATP via oxidative phosphorylation; (2) used for post-translational palmitoylation of target protein cysteine residues; (3) condensed with other molecules to produce triglycerides for energy storage and/or phospholipids for membrane biogenesis. Enzymes are indicated in blue. (D) Temporal traces of the DEAD box DNA helicases DDX1 and DDX46 relative protein abundances at the indicated DPI of primary human B-cell EBV infection. Data show the mean + SEM of n = 3 biological replicates.
(TIF)

S3 Fig Interplay between SREBP2, EBNA2 and MYC in LCL lipid biosynthesis gene regulation. (A) ChIP-seq tracks for the indicated transcription factors or H3K27Ac at the LCL *SREBF2* locus. Y-axis ranges are indicated for each track. (B) Mean + SEM of input versus day 21 *SREBF2*-targeting sgRNA abundances from genome-scale CRISPR/Cas9 screen performed in quadruplicate in Cas9+ GM12878 LCLs (71). Each sgRNA targets an independent *SREBF2* exon regions. The y-axis value refers to the log2-transformed number of reads for each sgRNA normalized to the total number of reads. (C) Mean + SEM of input versus day 21 *SREBF1*-targeting sgRNA abundances from genome-scale CRISPR/Cas9 screen performed in quadruplicate in Cas9+ GM12878 LCLs (71). Each sgRNA targets an independent *SREBF1* exon regions. The y-axis value refers to the log2-transformed number of reads for each sgRNA normalized to the total number of reads. (D) Dose-response curve analysis of fatostatin on newly-infected primary human B-cell growth and survival. Newly infected primary human B-cells were treated with the indicated doses of fatostatin or DMSO vehicle control for 4–7 DPI. The fatostatin effective concentration 50 (EC_50_) on newly-infected B-cell outgrowth was determined by GraphPad curve fitting analysis, as shown. (E) ChIP-seq tracks for the indicated transcription factors or H3K27Ac at the LCL *ACACA* locus, which encodes the ACC1 enzyme. The y-axis value refers to the log2-transformed number of reads for each sgRNA normalized to the total number of reads. (F) RT-PCR analysis of mRNAs encoding the fatty acid synthesis pathway enzymes ACLY or SCD, the cholesterol pathway enzymes HMGCR or FDFT1, LDLR, or the GGT-I subunits FNTA and PGGT1B from in primary human B-cells that were either mock-infected or infected with equal amounts of the non-transforming P3HR-1, UV-irradiated B95-8 or B95-8 EBV strains for four days. Mean values + SEM from n = 3 replicates are shown. *, p<0.05; **p, <0.01 (two-tailed t-test).
(TIF)

S4 Fig HMGCR and mevalonate pathway role EBV-infected cell outgrowth and survival. (A) Immunoblot analysis of whole cell lysates from Cas9+ GM12878 LCL expressing control or *HMGCR* targeting sgRNAs as indicated. (B) RT-PCR analysis of mRNAs encoding the cholesterol pathway enzymes FDFT1, SQLE, or LDLR from newly infected primary human B-cells treated for DPI 2–7 with the indicated doses of simvastatin or DMSO vehicle control. Mean values + SEM from n = 3 replicates are shown. *, p<0.05; **p, <0.01. (two tailed t-test). (C) Dose-response curve analysis of simvastatin on newly-infected primary human B-cell growth and survival. Shown are relative live cell numbers of EBV-infected primary human B-cells treated with the indicated doses of simvastatin or with DMSO vehicle control from day 4–7 post-infection. Mean + SEM values for n = 3 replicates are shown. The simvastatin effective concentration 50 (EC_50_) on newly-infected B-cell outgrowth was determined by GraphPad non-linear regression analysis, as shown. (D) Fold change in live cell number of primary human B-cells infected by EBV for the indicated DPI, cultured in the presence of DMSO or atorvastatin (2 μM) from 2 DPI to 7 DPI. Data show the mean + SEM from n = 3 replicates. *, p<0.05; **, p<0.01 (two-tailed t-test). (E) Mean + SEM of CFSE abundance values of primary human B-cells newly infected with B95-8 EBV or GM12878 LCL treated with DMSO, simvastatin (2 μM) and/or GGPP (2 μM), as indicated from days post-infection (DPI) 2–7, or from days in culture (DIC) 2–7 for GM12878 LCLs. Data were collected from n = 3 replicates, a representative one of which is shown in Fig 3E. (F) Mean + SEM of 7-AAD cell death abundance values of primary human B-cells newly infected with B95-8 EBV or GM12878 LCL treated with DMSO, simvastatin (2 μM) and/or GGPP (2 μM), as indicated from days post-infection (DPI) 2–7, or from days in culture (DIC) 2–7 for GM12878 LCLs. Data were collected from n = 3 replicates, a representative one of which is shown in Fig 3E. (G) RT-PCR analysis of mRNAs encoding the cholesterol pathway enzymes HMGCR, FDFT1, SQLE, or LDLR from newly infected primary human B-cells treated for DPI 2–7 with the indicated doses of simvastatin or DMSO vehicle control in the absence or presence of GGPP, as indicated. simvastatin and GGPP were refreshed at 4 DPI. Mean values + SEM from n = 3 replicates are shown. Shown as a control are mRNA abundances for the GGT-I subunits, which are not known to be altered by HMGCR blockade and which were not significantly changed by simvastatin treatment. *, p<0.05; **p, <0.01 (two-tailed t-test).
(TIF)

S5 Fig Key role of GGT-II in EBV infected B-cell growth and survival. (A) Immunoblot analysis of GGT-I target Rap1a/b and GAPDH load control levels in newly infected primary human B-cells treated with the indicated concentrations of GGT-I inhibitor GGTI-2133 (GGT-Ii) or DMSO vehicle control DPI 4–7. Loss of prenylation causes higher Rap1a/b electrophoretic mobility, indicated by the *. (B) Immunoblot analysis of GGT-II target Rab13, LMP1 target TRAF1, phospho-mTor S2448 and GAPDH load control abundances in newly infected primary human B-cells treated with the indicated doses of GGT-IIi inhibitor BMS-214662 or DMSO control for DPI 4–7. Loss of prenylation causes higher Rab13 electrophoretic mobility, indicated by the *. (C) Dose-response curve analysis of GGT-I inhibitor GGTI-2133 on newly-infected primary human B-cell growth and survival. Shown are relative live cell numbers of EBV-infected primary human B-cells treated with the indicated doses of GGTI-2133 or with DMSO vehicle control from day 4–7 post-infection. An effective concentration 50 (EC_50_) on newly-infected B-cell outgrowth not determined. Mean + SEM values for three replicates are shown. (D) Dose-response curve analysis of GGT-II inhibitor BMS-214662 on newly-infected primary human B-cell growth and survival. Shown are relative live cell numbers of EBV-infected primary human B-cells treated with the indicated doses of BMS-214662 or with DMSO vehicle control from day 4–7 post-infection. Mean + SEM values for three replicates are shown. EC_50_ on newly-infected B-cell outgrowth was determined by GraphPad non-linear regression analysis. (E-F) Mean + SEM of input versus day 21 abundances of *FNTA*-targeting (left) or *PGGT1B*-targeting sgRNAs from a genome-scale CRISPR/Cas9 screen performed in quadruplicate in Cas9+ GM12878 LCLs (71). *FNTA* and *PGGT1B* encode GGT-I subunits. Each sgRNA targets independent exon regions. The y-axis value refers to the log2-transformed number of reads for each sgRNA normalized to the total number of reads. (G-H) Mean + SEM of input versus day 21 *RABGGTA*-targeting (G) or *RABGGTB*-targeting (H) sgRNA abundances from genome-scale CRISPR/Cas9 screen performed in quadruplicate in Cas9+ GM12878 LCLs (71). *RABGGTA* and *RABGGTB* encode GGT-II subunits. Each sgRNA targets an independent exon.
(TIF)

S6 Fig GGT-II role in activation of the kinases P85 and SYK in LCLs. (A-B) Immunoblot analysis of whole cell lysates from Cas9+ Gm12878 LCLs expressing non-targeting control or independent *RABGGTA* (A) or *RABGGTB* (B) targeting sgRNAs. (C) Propidium Iodide cell cycle analysis of conditional EBNA3C-HT 2-2-3 LCLs grown in the presence of 4HT (column 1) or in the absence of 4HT for 14 days (columns 2–4) and then treated with 4HT (1 μM) for 24 hours to re-induce EBNA3C expression, where indicated. Also where indicated, EBNA3C induction was performed in media lacking 10% fetal bovine serum (serum) in order to control for EBNA3C effects on cell cycle. See also Fig 5F.
(TIF)

S7 Fig EBV-induced Rab13 co-localizes with LMP2A in newly infected primary human B-cells. (A) Immunofluorescence micrographs of LMP2A, Rab13 and nuclear Hoechst 33528 uninfected versus infected primary human B-cells at 4 DPI. Merged images are shown with white boxes indicating inset images that have been further magnified. Scale bar is indicated (50 μm for single-channel and merged images). Three columns are presented for DPI 4 in order to increase the number of cells displayed at this timepoint. See also S6 Fig. (B) Mean + SEM of input versus day 21 *RAB13*-targeting sgRNA abundances from genome-scale CRISPR/Cas9 screen performed in quadruplicate in Cas9+ GM12878 LCLs (71). Each sgRNA targets an independent exon
(TIF)

S8 Fig Mevalonate pathway GGPP production is important for Rab13 and LMP2A trafficking in newly infected B-cells. Immunofluorescence micrographs of from newly infected primary B-cells cultured from days 2–7 post infection in the presence of DMSO, simvastatin (2 μM) or GGPP (2 μM) as indicated. Shown are images from n = 3 experiments. Scale bar is indicated (50 μm for single-channel and merged images). See also Fig 6B.
(TIF)

S9 Fig GGT-II is important forLMP2A and Rab13 trafficking in newly infected B-cells. Immunofluorescence micrographs of from newly infected primary B-cells cultured from days 4–5 post infection in the presence of DMSO or the indicated concentration of GGT-II inhibitor BMS-214662. Shown are images from n = 3 experiments. Scale bar is indicated (50 μm for single-channel and merged images). See also Fig 6B.
(TIF)

S10 Fig Rab13 is important for LCL LMP1 target gene induction, growth and survival. (A) Quantitative RT-PCR analysis of TRAF1 mRNA abundance in Cas9+ GM12878 LCL that express either control or an independent *RAB13*-targeting sgRNAs. LMP1 target *TRAF1* mRNA abundance was quantitated with *GAPDH* used for normalization. Data show the mean + SEM from n = 3 replicates. *, p<0.05 (one-sample t-test). (B) Immunoblot analysis of LMP1 and LMP2A from WCL of Cas9+ GM12878 LCL that express either control or independent *RAB13*-targeting sgRNAs. Representative blots from n = 2 experiments are shown.
(TIF)

S1 Table GSEA analysis of metabolism-related proteins detected in whole cell proteomic analysis of primary human B-cells infected by EBV. (XLSX)

S2 Table List of proteins from enriched pathways identified by the GSEA analysis shown in S1 Table. (XLSX)

S3 Table Sequences of CRISPR sgRNAs used in this study. (XLSX)

S4 Table Sequences of primers used in this study. (XLSX)
