# Supplementary figures and images for: Epstein-Barr virus subverts mevalonate and fatty acid pathways to promote infected B-cell proliferation and survival
Source: PLoS Pathog. 2019 Sep 13;15(9):e1008030. doi: 10.1371/journal.ppat.1008030 (PMC6760809; doi:10.1371/journal.ppat.1008030)

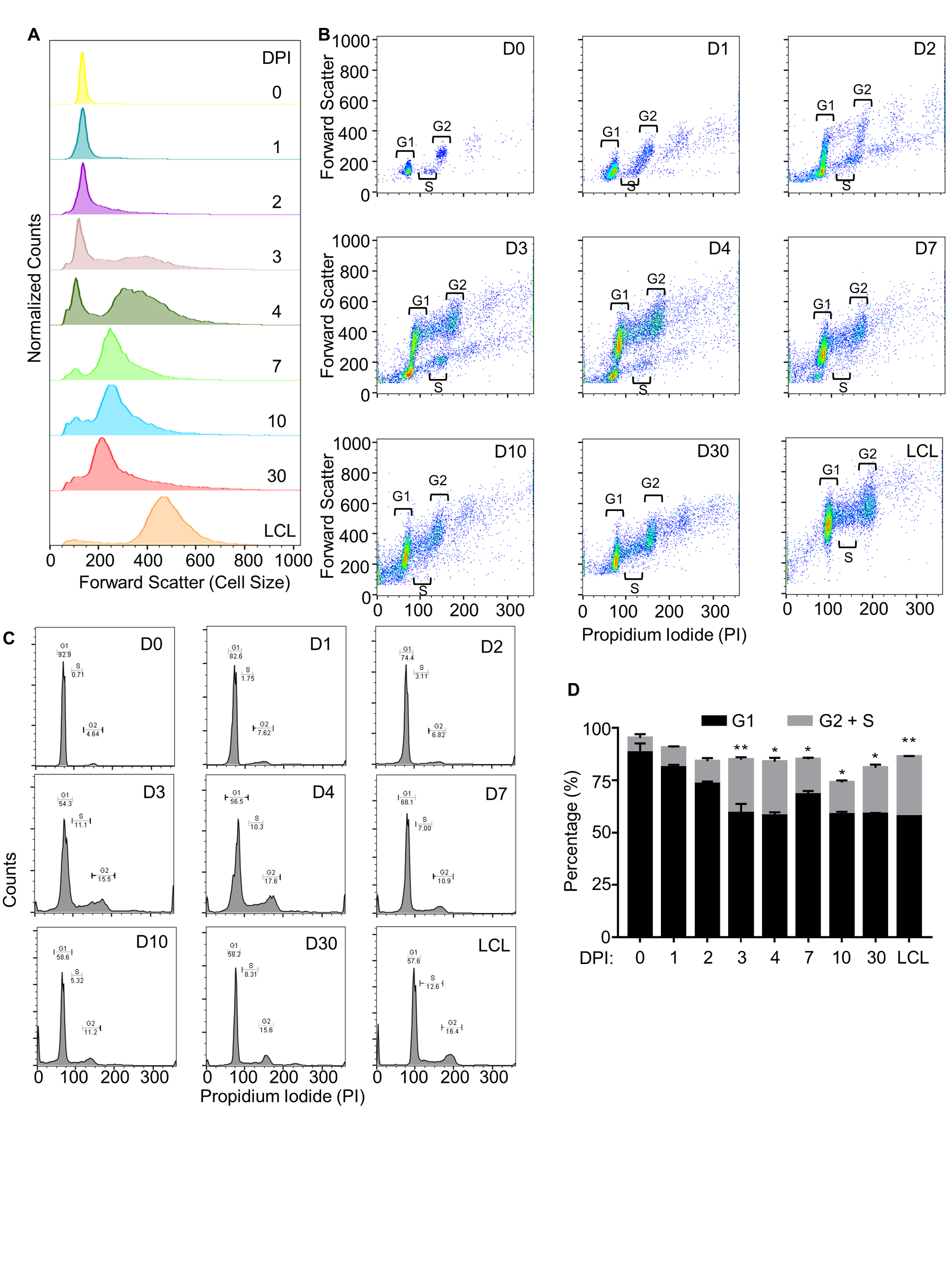

Supplement: S1 Fig — (A) FACS forward scatter cell size measurements of live CD19+ primary human B-cells at the indicated days post-infection (DPI) by B95-8 EBV at a MOI of 0.1. Shown is a representative experiment of n = 3 replicates. Of note, cells were not gated for CD23 as a marker of EBV infection. (B) Propidium iodide (PI) cell cycle and forward scatter cell size FACS analysis are shown for the indicated DPI timepoints from the experiment shown in panel S1A. Data were representative of n = 3 experiments. (C) Propidium iodide (PI) cell cycle analysis for the indicated DPI timepoints from the experiment shown in panels S1A-B. Data were representative of n = 3 experiments. (D) Mean +SEM values of the numbers of cell identified as G1 or as in either G2 or S phases (G2+S) of the cell cycle by the PI analysis at the indicated DPI timepoints from n = 3 replicates, including the experiment presented in panels S1A-C. *, p<0.05; **, p<0.01. (two-tailed t-test). (TIF) [file ppat.1008030.s001.tif]

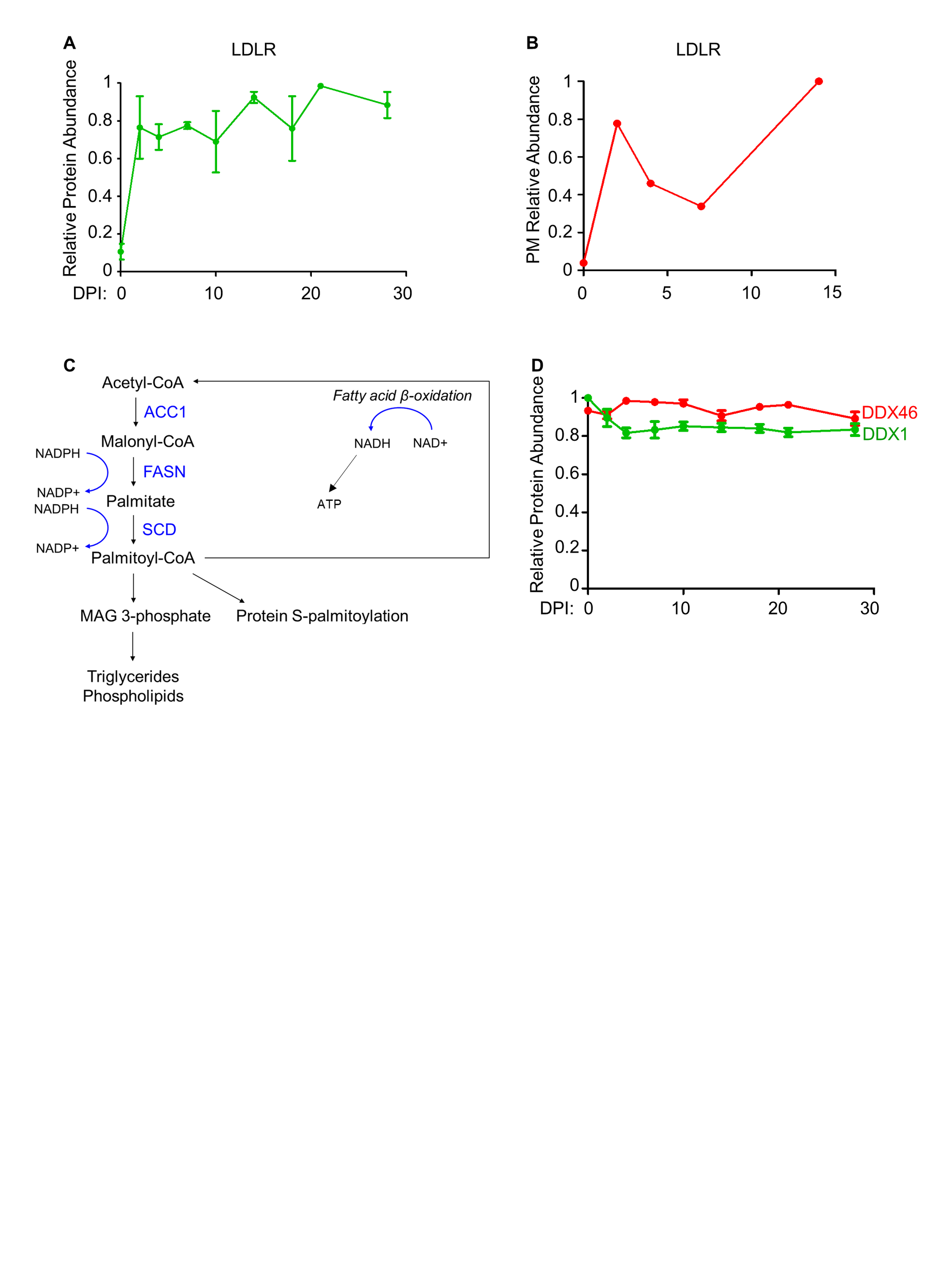

Supplement: S2 Fig — (A) Temporal traces of whole cell LDL receptor (LDLR) relative protein abundances at the indicated DPI of primary human B-cell EBV infection. Data show the mean + SEM of n = 3 biological replicates. (B) Temporal traces of plasma membrane (PM) LDLR relative protein abundances at the indicated DPI of primary human B-cell EBV infection. (C) Schematic diagram showing de novo lipid synthesis pathway conversion of glucose-derived acetyl-CoA into end products. NADPH-dependent acetyl-CoA reduction produces palmitate, which can be directed to one of three routes: (1) oxidation via the fatty acid β-oxidation pathway to produce reducing power in the form of NADH and ultimately, ATP via oxidative phosphorylation; (2) used for post-translational palmitoylation of target protein cysteine residues; (3) condensed with other molecules to produce triglycerides for energy storage and/or phospholipids for membrane biogenesis. Enzymes are indicated in blue. (D) Temporal traces of the DEAD box DNA helicases DDX1 and DDX46 relative protein abundances at the indicated DPI of primary human B-cell EBV infection. Data show the mean + SEM of n = 3 biological replicates. (TIF) [file ppat.1008030.s002.tif]

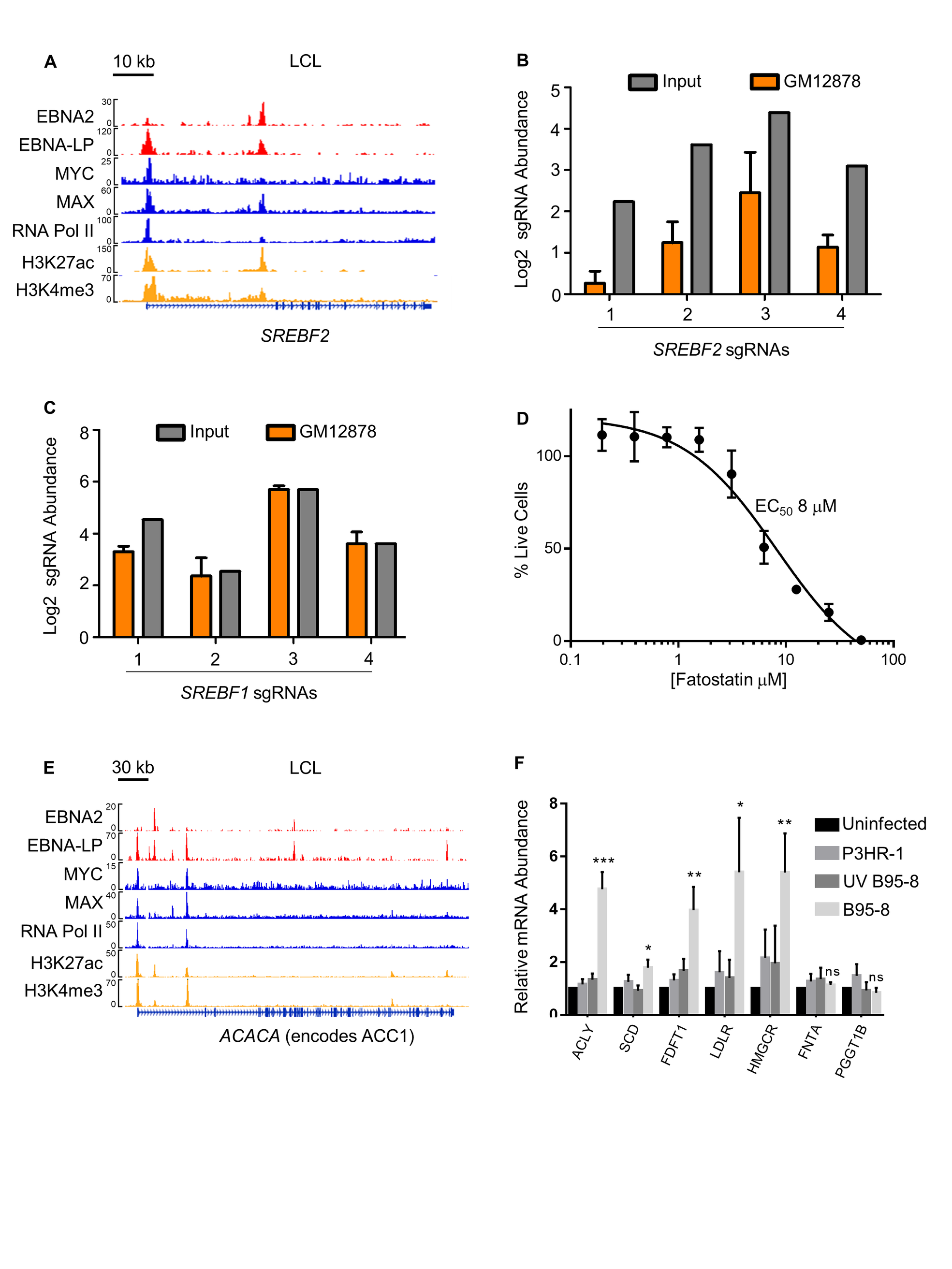

Supplement: S3 Fig — (A) ChIP-seq tracks for the indicated transcription factors or H3K27Ac at the LCL SREBF2 locus. Y-axis ranges are indicated for each track. (B) Mean + SEM of input versus day 21 SREBF2-targeting sgRNA abundances from genome-scale CRISPR/Cas9 screen performed in quadruplicate in Cas9+ GM12878 LCLs (71). Each sgRNA targets an independent SREBF2 exon regions. The y-axis value refers to the log2-transformed number of reads for each sgRNA normalized to the total number of reads. (C) Mean + SEM of input versus day 21 SREBF1-targeting sgRNA abundances from genome-scale CRISPR/Cas9 screen performed in quadruplicate in Cas9+ GM12878 LCLs (71). Each sgRNA targets an independent SREBF1 exon regions. The y-axis value refers to the log2-transformed number of reads for each sgRNA normalized to the total number of reads. (D) Dose-response curve analysis of fatostatin on newly-infected primary human B-cell growth and survival. Newly infected primary human B-cells were treated with the indicated doses of fatostatin or DMSO vehicle control for 4–7 DPI. The fatostatin effective concentration 50 (EC50) on newly-infected B-cell outgrowth was determined by GraphPad curve fitting analysis, as shown. (E) ChIP-seq tracks for the indicated transcription factors or H3K27Ac at the LCL ACACA locus, which encodes the ACC1 enzyme. The y-axis value refers to the log2-transformed number of reads for each sgRNA normalized to the total number of reads. (F) RT-PCR analysis of mRNAs encoding the fatty acid synthesis pathway enzymes ACLY or SCD, the cholesterol pathway enzymes HMGCR or FDFT1, LDLR, or the GGT-I subunits FNTA and PGGT1B from in primary human B-cells that were either mock-infected or infected with equal amounts of the non-transforming P3HR-1, UV-irradiated B95-8 or B95-8 EBV strains for four days. Mean values + SEM from n = 3 replicates are shown. *, p<0.05; **p, <0.01 (two-tailed t-test). (TIF) [file ppat.1008030.s003.tif]

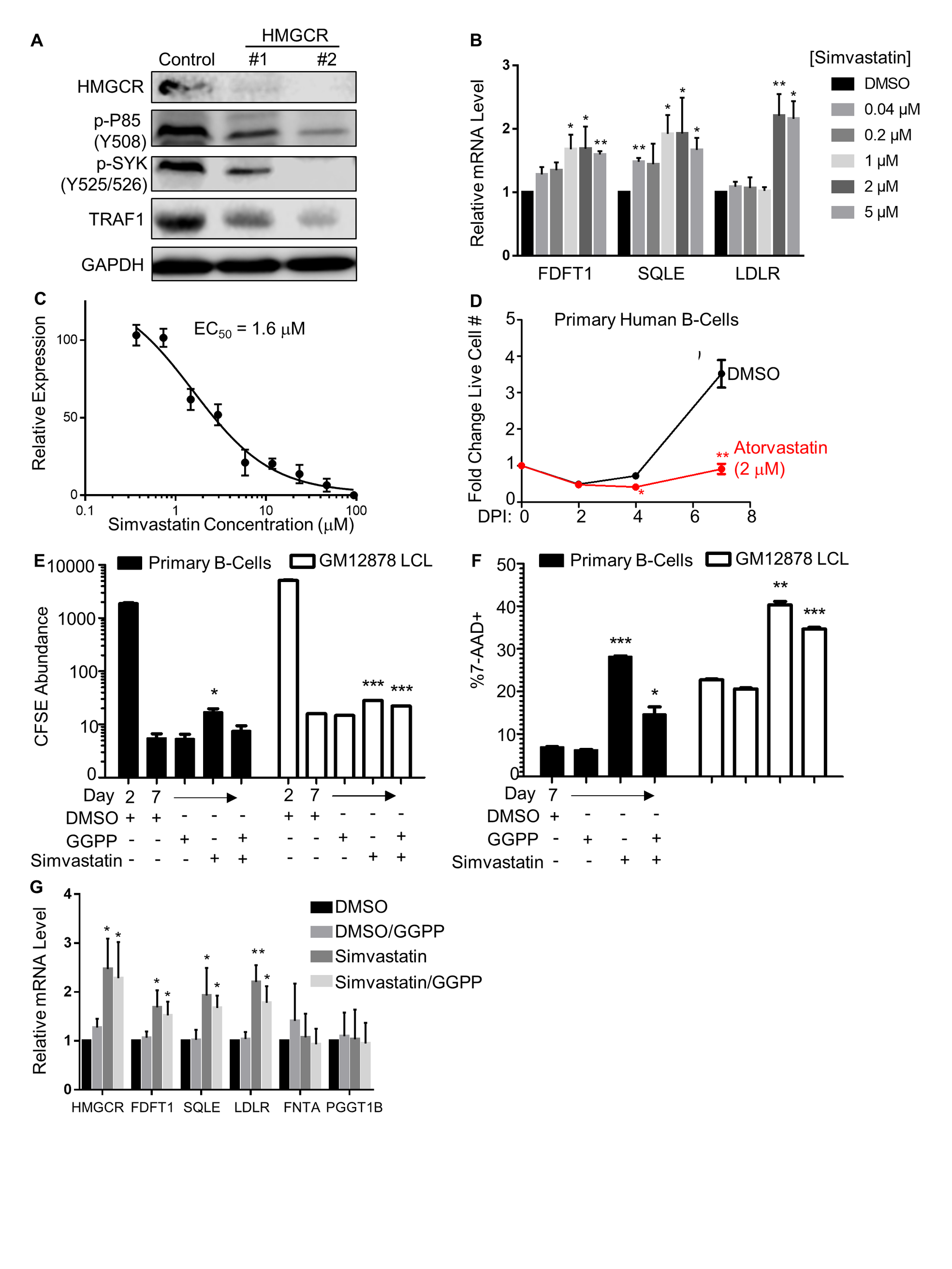

Supplement: S4 Fig — (A) Immunoblot analysis of whole cell lysates from Cas9+ GM12878 LCL expressing control or HMGCR targeting sgRNAs as indicated. (B) RT-PCR analysis of mRNAs encoding the cholesterol pathway enzymes FDFT1, SQLE, or LDLR from newly infected primary human B-cells treated for DPI 2–7 with the indicated doses of simvastatin or DMSO vehicle control. Mean values + SEM from n = 3 replicates are shown. *, p<0.05; **p, <0.01. (two tailed t-test). (C) Dose-response curve analysis of simvastatin on newly-infected primary human B-cell growth and survival. Shown are relative live cell numbers of EBV-infected primary human B-cells treated with the indicated doses of simvastatin or with DMSO vehicle control from day 4–7 post-infection. Mean + SEM values for n = 3 replicates are shown. The simvastatin effective concentration 50 (EC50) on newly-infected B-cell outgrowth was determined by GraphPad non-linear regression analysis, as shown. (D) Fold change in live cell number of primary human B-cells infected by EBV for the indicated DPI, cultured in the presence of DMSO or atorvastatin (2 μM) from 2 DPI to 7 DPI. Data show the mean + SEM from n = 3 replicates. *, p<0.05; **, p<0.01 (two-tailed t-test). (E) Mean + SEM of CFSE abundance values of primary human B-cells newly infected with B95-8 EBV or GM12878 LCL treated with DMSO, simvastatin (2 μM) and/or GGPP (2 μM), as indicated from days post-infection (DPI) 2–7, or from days in culture (DIC) 2–7 for GM12878 LCLs. Data were collected from n = 3 replicates, a representative one of which is shown in Fig 3E. (F) Mean + SEM of 7-AAD cell death abundance values of primary human B-cells newly infected with B95-8 EBV or GM12878 LCL treated with DMSO, simvastatin (2 μM) and/or GGPP (2 μM), as indicated from days post-infection (DPI) 2–7, or from days in culture (DIC) 2–7 for GM12878 LCLs. Data were collected from n = 3 replicates, a representative one of which is shown in Fig 3E. (G) RT-PCR analysis of mRNAs encoding the cholesterol pathway [file ppat.1008030.s004.tif]

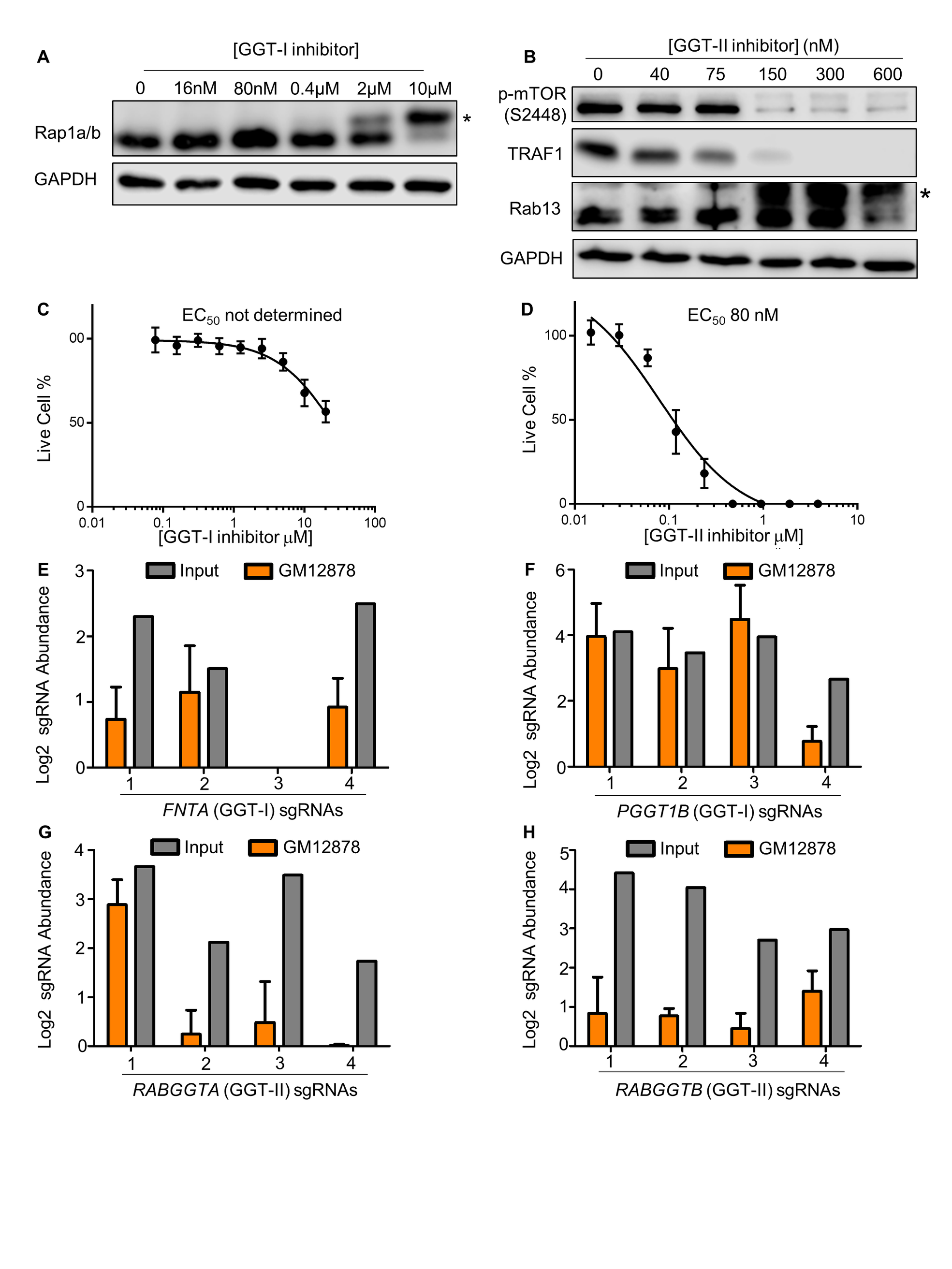

Supplement: S5 Fig — (A) Immunoblot analysis of GGT-I target Rap1a/b and GAPDH load control levels in newly infected primary human B-cells treated with the indicated concentrations of GGT-I inhibitor GGTI-2133 (GGT-Ii) or DMSO vehicle control DPI 4–7. Loss of prenylation causes higher Rap1a/b electrophoretic mobility, indicated by the *. (B) Immunoblot analysis of GGT-II target Rab13, LMP1 target TRAF1, phospho-mTor S2448 and GAPDH load control abundances in newly infected primary human B-cells treated with the indicated doses of GGT-IIi inhibitor BMS-214662 or DMSO control for DPI 4–7. Loss of prenylation causes higher Rab13 electrophoretic mobility, indicated by the *. (C) Dose-response curve analysis of GGT-I inhibitor GGTI-2133 on newly-infected primary human B-cell growth and survival. Shown are relative live cell numbers of EBV-infected primary human B-cells treated with the indicated doses of GGTI-2133 or with DMSO vehicle control from day 4–7 post-infection. An effective concentration 50 (EC50) on newly-infected B-cell outgrowth not determined. Mean + SEM values for three replicates are shown. (D) Dose-response curve analysis of GGT-II inhibitor BMS-214662 on newly-infected primary human B-cell growth and survival. Shown are relative live cell numbers of EBV-infected primary human B-cells treated with the indicated doses of BMS-214662 or with DMSO vehicle control from day 4–7 post-infection. Mean + SEM values for three replicates are shown. EC50 on newly-infected B-cell outgrowth was determined by GraphPad non-linear regression analysis. (E-F) Mean + SEM of input versus day 21 abundances of FNTA-targeting (left) or PGGT1B-targeting sgRNAs from a genome-scale CRISPR/Cas9 screen performed in quadruplicate in Cas9+ GM12878 LCLs (71). FNTA and PGGT1B encode GGT-I subunits. Each sgRNA targets independent exon regions. The y-axis value refers to the log2-transformed number of reads for each sgRNA normalized to the total number of reads. (G-H) Mean + SEM of input versus day 21 RABGGTA- [file ppat.1008030.s005.tif]

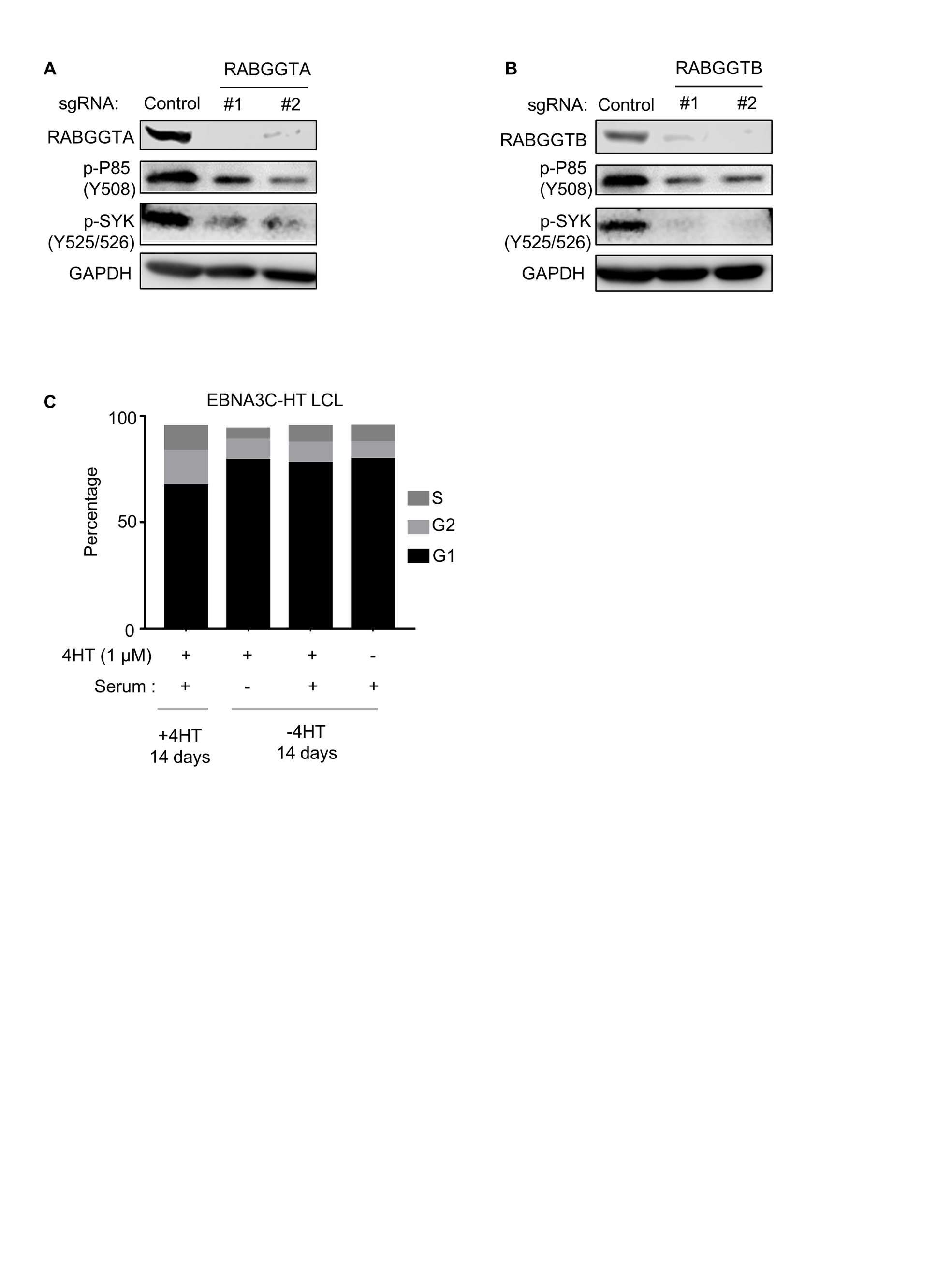

Supplement: S6 Fig — (A-B) Immunoblot analysis of whole cell lysates from Cas9+ Gm12878 LCLs expressing non-targeting control or independent RABGGTA (A) or RABGGTB (B) targeting sgRNAs. (C) Propidium Iodide cell cycle analysis of conditional EBNA3C-HT 2-2-3 LCLs grown in the presence of 4HT (column 1) or in the absence of 4HT for 14 days (columns 2–4) and then treated with 4HT (1 μM) for 24 hours to re-induce EBNA3C expression, where indicated. Also where indicated, EBNA3C induction was performed in media lacking 10% fetal bovine serum (serum) in order to control for EBNA3C effects on cell cycle. See also Fig 5F. (TIF) [file ppat.1008030.s006.tif]

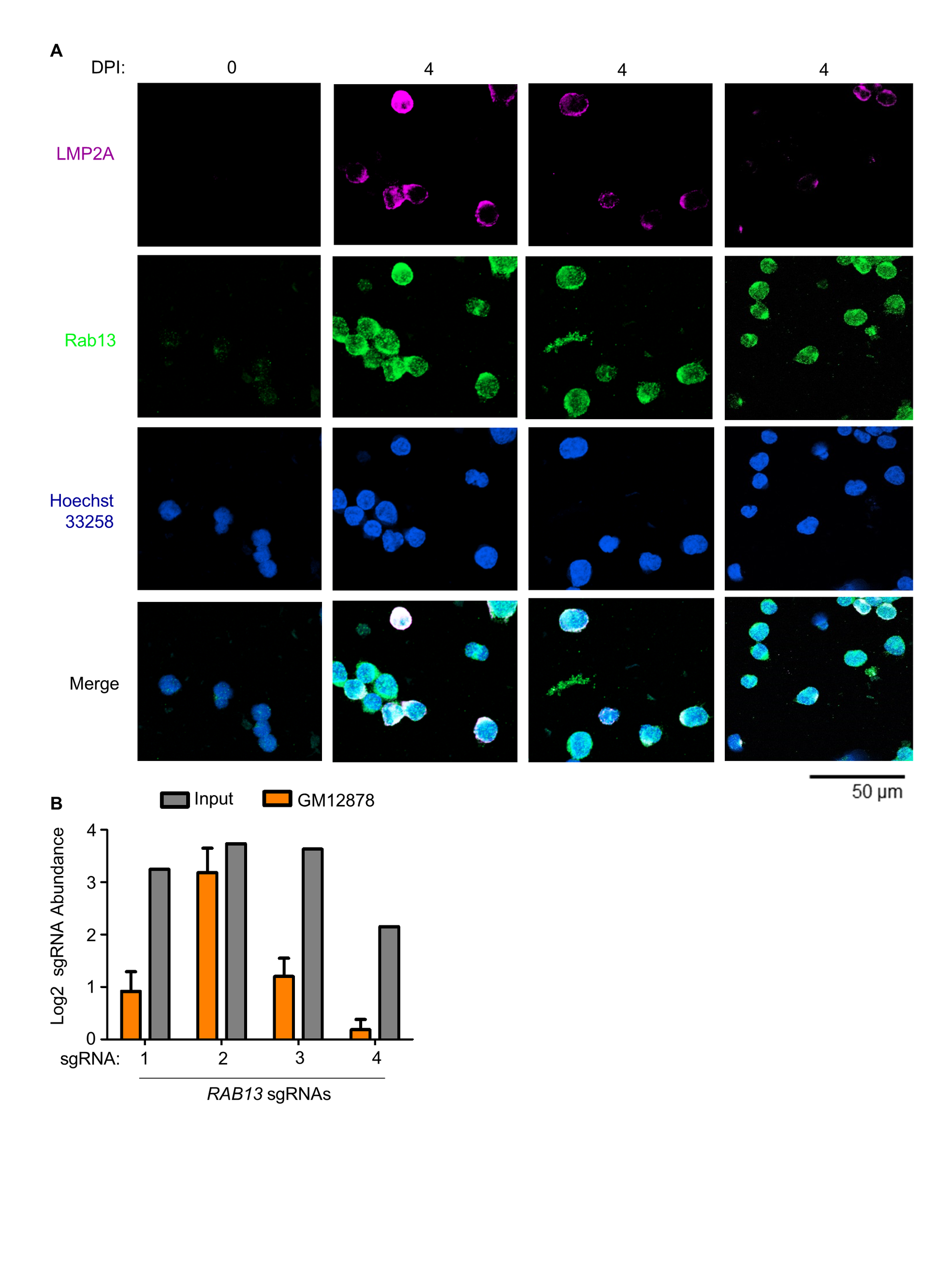

Supplement: S7 Fig — (A) Immunofluorescence micrographs of LMP2A, Rab13 and nuclear Hoechst 33528 uninfected versus infected primary human B-cells at 4 DPI. Merged images are shown with white boxes indicating inset images that have been further magnified. Scale bar is indicated (50 μm for single-channel and merged images). Three columns are presented for DPI 4 in order to increase the number of cells displayed at this timepoint. See also S6 Fig. (B) Mean + SEM of input versus day 21 RAB13-targeting sgRNA abundances from genome-scale CRISPR/Cas9 screen performed in quadruplicate in Cas9+ GM12878 LCLs (71). Each sgRNA targets an independent exon (TIF) [file ppat.1008030.s007.tif]

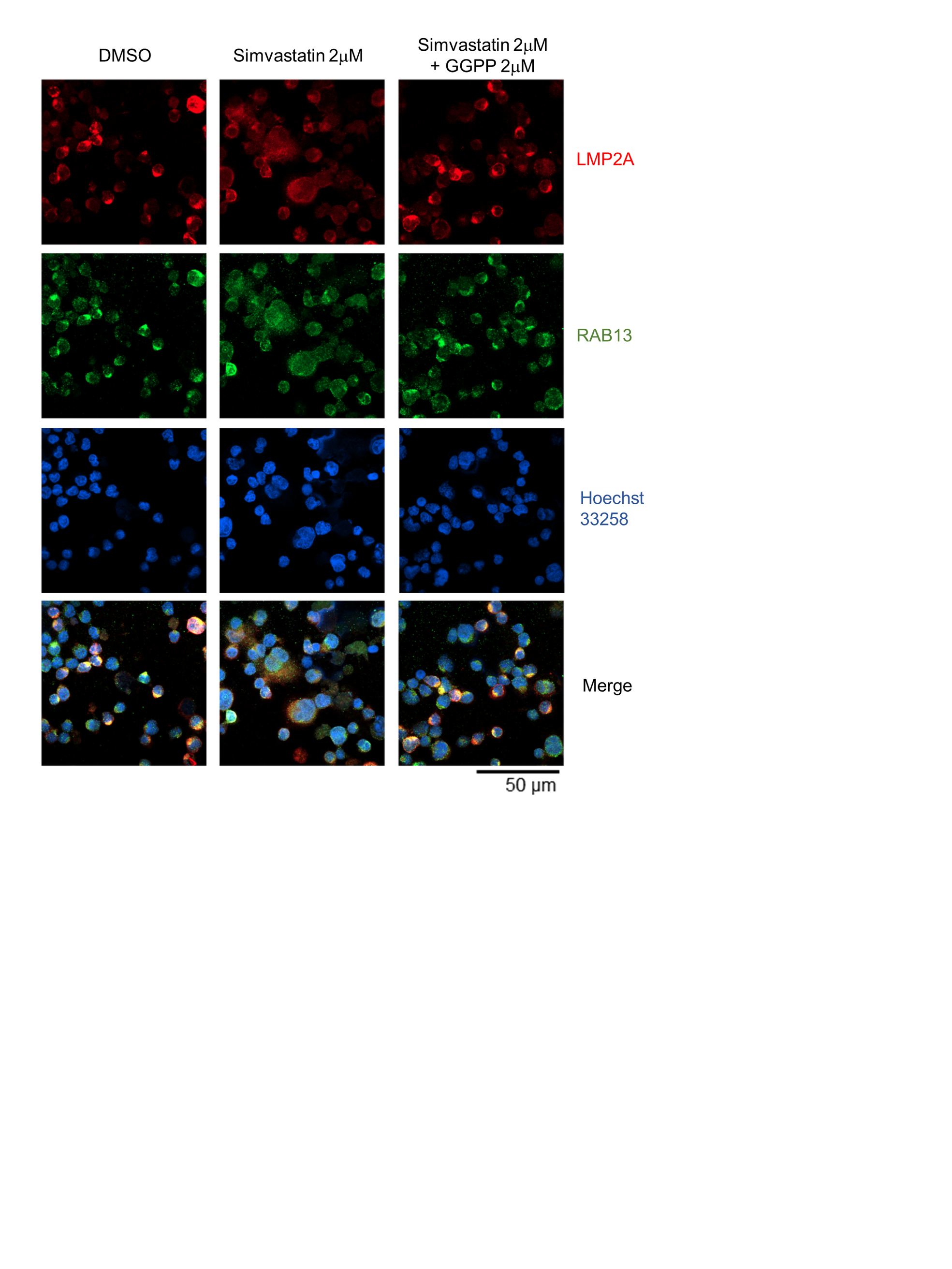

Supplement: S8 Fig — Immunofluorescence micrographs of from newly infected primary B-cells cultured from days 2–7 post infection in the presence of DMSO, simvastatin (2 μM) or GGPP (2 μM) as indicated. Shown are images from n = 3 experiments. Scale bar is indicated (50 μm for single-channel and merged images). See also Fig 6B. (TIF) [file ppat.1008030.s008.tif]

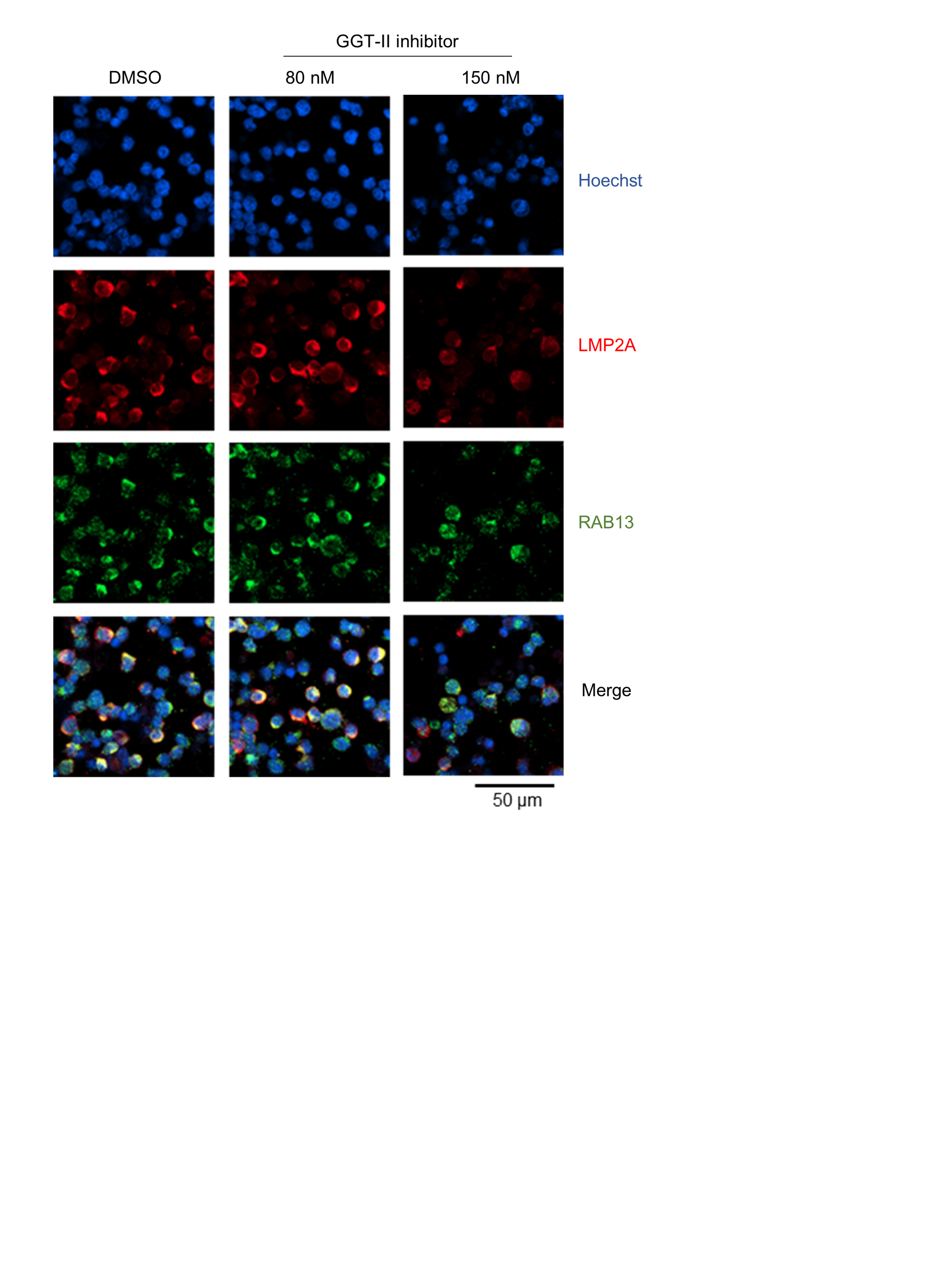

Supplement: S9 Fig — Immunofluorescence micrographs of from newly infected primary B-cells cultured from days 4–5 post infection in the presence of DMSO or the indicated concentration of GGT-II inhibitor BMS-214662. Shown are images from n = 3 experiments. Scale bar is indicated (50 μm for single-channel and merged images). See also Fig 6B. (TIF) [file ppat.1008030.s009.tif]

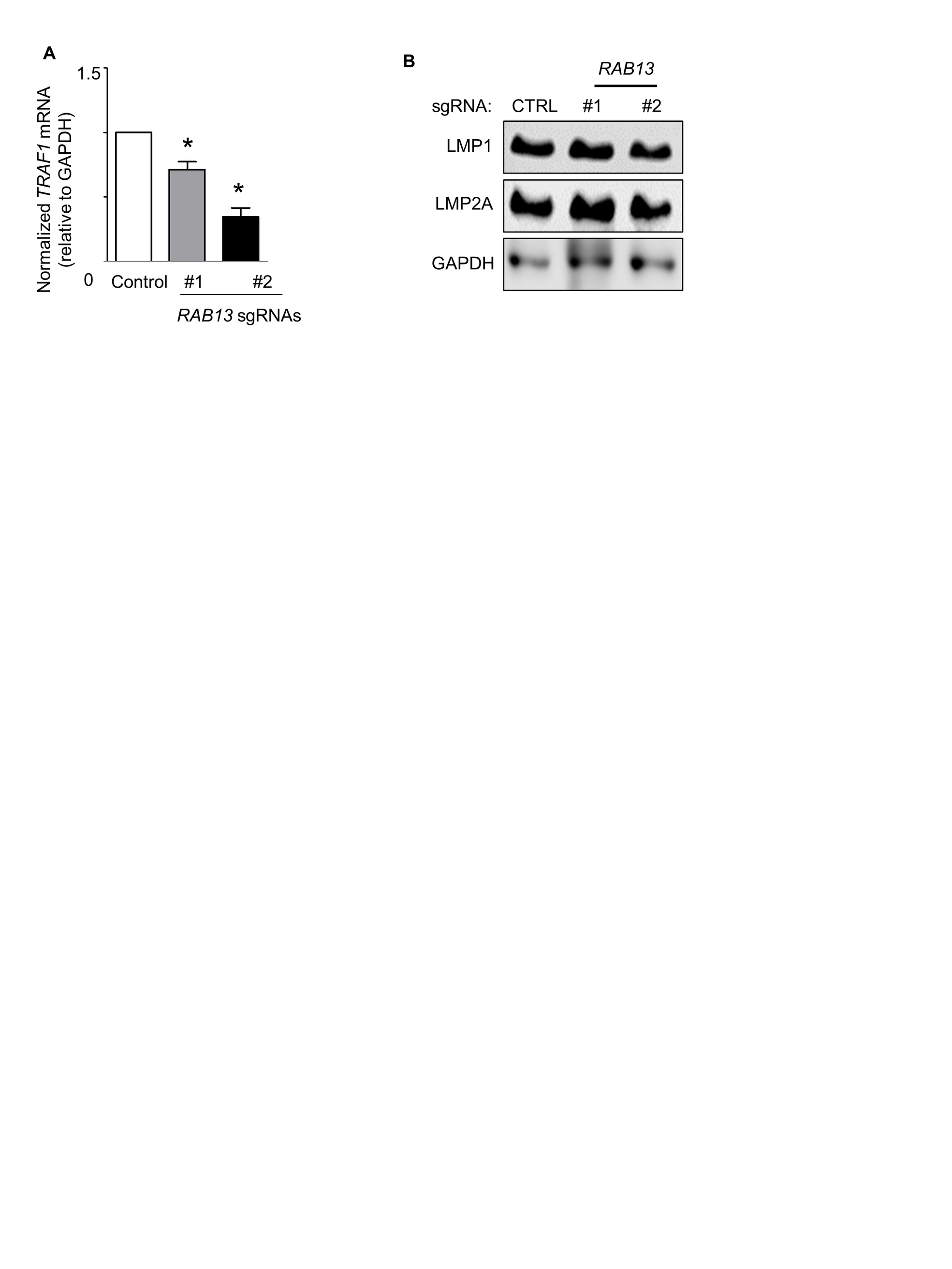

Supplement: S10 Fig — (A) Quantitative RT-PCR analysis of TRAF1 mRNA abundance in Cas9+ GM12878 LCL that express either control or an independent RAB13-targeting sgRNAs. LMP1 target TRAF1 mRNA abundance was quantitated with GAPDH used for normalization. Data show the mean + SEM from n = 3 replicates. *, p<0.05 (one-sample t-test). (B) Immunoblot analysis of LMP1 and LMP2A from WCL of Cas9+ GM12878 LCL that express either control or independent RAB13-targeting sgRNAs. Representative blots from n = 2 experiments are shown. (TIF) [file ppat.1008030.s010.tif]
